# The telomere-to-telomere gapless genome of grass carp provides insights for genetic improvement

**DOI:** 10.1093/gigascience/giaf059

**Published:** 2025-06-18

**Authors:** Fei Liu, Yuan Li, Guishuang Wang, Dong Zhang, Xinlan Yang, Chaowei Zhou, Rongzhu Zhou, Haiping Liu

**Affiliations:** School of Ecology and Environment, Tibet University, Lhasa, Tibet 850000, China; Institute of Aquatic Sciences, Tibet Autonomous Region Academy of Agricultural and Animal Husbandry Sciences, Lhasa, Tibet 850000, China; Integrative Science Center of Germplasm Creation in Western China (CHONGQING) Science City, Key Laboratory of Freshwater Fish Reproduction and Development (Ministry of Education), Key Laboratory of Chongqing Municipality for Aquatic Economic Animal Resources Conservation and Germplasm Creation, College of Fisheries, Southwest University, Chongqing 400715, China; Wuhan Huabiology Co., Ltd., Wuhan, Hubei 430000, China; Institute of Aquatic Sciences, Tibet Autonomous Region Academy of Agricultural and Animal Husbandry Sciences, Lhasa, Tibet 850000, China; School of Ecology and Environment, Tibet University, Lhasa, Tibet 850000, China; Institute of Aquatic Sciences, Tibet Autonomous Region Academy of Agricultural and Animal Husbandry Sciences, Lhasa, Tibet 850000, China; Institute of Aquatic Sciences, Tibet Autonomous Region Academy of Agricultural and Animal Husbandry Sciences, Lhasa, Tibet 850000, China; Integrative Science Center of Germplasm Creation in Western China (CHONGQING) Science City, Key Laboratory of Freshwater Fish Reproduction and Development (Ministry of Education), Key Laboratory of Chongqing Municipality for Aquatic Economic Animal Resources Conservation and Germplasm Creation, College of Fisheries, Southwest University, Chongqing 400715, China; National Animal Husbandry Services, Beijing 100125, China; School of Ecology and Environment, Tibet University, Lhasa, Tibet 850000, China; Integrative Science Center of Germplasm Creation in Western China (CHONGQING) Science City, Key Laboratory of Freshwater Fish Reproduction and Development (Ministry of Education), Key Laboratory of Chongqing Municipality for Aquatic Economic Animal Resources Conservation and Germplasm Creation, College of Fisheries, Southwest University, Chongqing 400715, China

**Keywords:** grass carp, telomere-to-telomere genome, phylogenomic

## Abstract

**Background:**

The grass carp (*Ctenopharyngodon idella*) is a large herbivorous freshwater fish belonging to the Cyprinidae family. It is widely cultivated as a food source in China and is renowned as one of the Four Great Domestic Fishes. Despite its economic importance, the published genome assemblies of grass carp remain incomplete due to gaps, thereby hindering molecular research and genetic improvement.

**Results:**

In this study, we report the assembly of a telomere-to-telomere (T2T) gap-free genome of the grass carp with total length of 890,918,310 bp for 24 chromosomes without gaps, representing the highest completeness and assembly quality to date. Our assembly contains 27,446 protein-coding genes, and 93.04% of all were annotated with multiple databases, with 48 telomeres and 24 centromeres characterized. Gap-free reference genomes enable us to study the structure of centromeres and identify conserved centromere-specific satellite motifs for grass carp. Furthermore, we identified 108 gene-related gaps across 12 chromosomes and 38 structural variations across 17 chromosomes in this T2T assembly.

**Conclusions:**

The validated gap-free genome provides invaluable resource for future genomic studies grass carp, offering new insights into its genetic architecture and evolutionary dynamics.

## Introduction

Grass carp (*Ctenopharyngodon idella*) (NCBI:txid7959; marinespecies.org:taxname:154314), a prominent member of the subfamily Leuciscinae in the Cyprinidae family, is distinguished by its adaptability, rapid growth, and large size [1, 2]. Its broad temperature tolerance has enabled its widespread distribution, particularly in the Yangtze, Pearl, and Heilongjiang river basins in China [3]. Recognized as one of China’s “Four Domesticated Fish” in freshwater aquaculture, grass carp has a rich history of over 1,700 years of cultivation within China. Since the 1980s, its aquaculture has expanded internationally to countries such as the United States, Mexico, India, and Hungary, establishing it as a valuable species in global aquaculture [4]. Grass carp also holds considerable economic importance, serving as an abundant source of high-quality protein and essential nutrients [3]. By 2023, global production of grass carp had reached 5.94 million tons, making it the most extensively farmed freshwater fish both in China and worldwide.

One of the few herbivorous species in freshwater aquaculture, grass carp is uniquely adapted to a plant-based diet, a dietary trait essential to its impressive growth and adaptive success [5]. During its transition from larvae to herbivorous adults, grass carp undergo significant increases in body weight, length, and intestinal length [5]. These physiological changes correspond with genetic adaptations involving circadian rhythms, lipid synthesis, and metabolic pathways, which enhance nutrient absorption and utilization from plant-based sources. These adaptive traits underscore the importance of grass carp in aquaculture and suggest potential avenues for further research into its genetic and physiological mechanisms for growth and nutrient efficiency [1, 6].

With advances in long-read whole-genome sequencing technologies such as PacBio HiFi and Oxford Nanopore, along with the continuous development and refinement of genome assembly software, many plants and animals—such as *Homo sapiens* [7, 8], *Zea mays* [9], and *Oryza sativa* [10]—have now achieved chromosome-level telomere-to-telomere (T2T) genome assemblies. Several fish species, including *Mastacembelus armatus* [11], *Clarias gariepinus* [12], and*Rhinogobio nasutus* [13], have also been sequenced to a chromosome-level T2T genome assembly. Although the grass carp genome was assembled using PacBio HiFi reads combined with high-throughput chromatin conformation (Hi-C) data, achieving a contig N50 of 19.3 Mb [14], a fully complete, high-quality chromosome-level genome assembly remains unavailable. Such a resource would be invaluable for advancing the study of biological functions, trait selection, and evolutionary research in this species.

In this study, we generated a T2T reference genome assembly of grass carp using DNB-T7 short reads, PacBio HiFi long reads, Hi-C technology reads, and Oxford Nanopore Technologies (ONT) ultra-long reads. We compared our assembly with the latest published grass carp genome versions, highlighting the differences and improvements, and conducted a detailed analysis of centromeric regions. Comparative genomic analysis with 11 other species allowed us to identify species-specific genes. This comprehensive, chromosome-scale genome provides a robust foundation for future research into grass carp genetics, functional gene discovery, and the evolutionary genomics of teleost fishes.

## Methods

### Sample collection and sequencing

A wild male *C. idella* collected from Hunan Fisheries Science Institute, Changsha, Hunan, China, was used in construction of the reference genome. Genomic DNA of *C. idella* was extracted from muscle tissue using the cetyltrimethylammonium bromide (CTAB) method for sequencing library construction. Following the standard protocols of the Pacific Biosciences, DNA libraries for single-molecule real-time PacBio genome sequencing were constructed, and circular consensus sequencing was performed using a PacBio Sequel IIe platform (RRID:SCR_017990) for high-fidelity (HiFi) reads. ONT ultra-long libraries were constructed and sequenced on an Oxford Nanopore promethION platform (RRID:SCR_017987) for ultra-long reads. Short-read libraries of *C. idella* were constructed according to the BGI DNBSEQ-T7 (RRID:SCR_017981) standard protocol, and paired-end reads (2 × 150 bp) were sequenced on a DNB-T7 platform. With default parameters, raw PacBio subreads were filtered and corrected using the pbccs pipeline.

A Hi-C library was constructed using muscle tissue of *C. idella*, which was fixed in 1% formaldehyde for crosslinking. Cells were lysed using a Dounce homogenizer and digested using the Hind III restriction enzyme. The DNA ends were filled and labeled with biotin, and the filled-in Hind III sites were ligated to form Nhe I sites. Complexes with the biotin-labeled ligation products were purified and sheared, and the biotinylated Hi-C ligation products were pulled down and used to construct a Hi-C library to obtain 2 × 150-bp paired-end reads using BGI DNBSEQ-T7.

### Genome assembly

The HiFi, ultra-long ONT, and Hi-C reads were integrated to produce the T2T assembly using Hifiasm (v. 0.19.9-r616, RRID:SCR_021069) with the parameters of –ul and verkko (v. 2.2) with default parameters, respectively [15, 16]. The HiFi reads and Hi-C reads were also assembled using Hifiasm with default parameters. The ultra-long ONT reads were assembled using nextDenovo (v. 2.5.2, RRID:SCR_025033) with parameters (read_cutoff = 1k, blocksize = 1 g, nextgraph_options = -a 1) [17]. These assemblies were evaluated, and the best primary assembly generated by Hifiasm (HiFi + ONT + Hi-C) was selected and then subsequently anchored onto chromosomes using Hi-C reads. To further obtain the haplotype-resolved genome, the haplotype assemblies from verkko (HiFi + ONT + HIC) were also selected for scaffolding using Hi-C reads.

Contig sequences were clustered into 24 chromosomal groups using ALLHiC (v. 0.9.8, RRID:SCR_022750) through agglomerative hierarchical clustering [18]. Within each group, contig sequencing and orientation were performed using ALLHi-C, followed by 3D-DNA (v. 180419, RRID:SCR_017227) [19] and Juicer (v. 1.6, RRID:SCR_017226) [20] to convert interaction data into binary files. Manual adjustments were carried out using Juicebox (v. 1.11.08, RRID:SCR_021172) [21].

Telomere repair was conducted using Winnowmap (v. 1.11, RRID:SCR_025349) [22], aligning ONT reads to the reference genome and focusing on reads within 50 bp of chromosome ends. Telomeric repeats (CCCTAA/TTAGGG) were identified, and the most frequent read was designated as the reference for Medaka consensus reassembly. The consensus was aligned to chromosomes using MUMmer (v. 3.1, RRID:SCR_018171) and replaced at the chromosome ends if identity exceeded 80% [23]. Gap filling was accomplished using Winnowmap to align gap-filling data (in the order of other assemblies > ONT reads > HiFi reads) to regions containing Ns in the genome, and error correction was performed on filled gap regions by Winnowmap [24] using HiFi reads (≥10 kb). Finally, we obtained the primary and 2 haplotype T2T assembles, as well as evaluated these genomes using different methods, including BUSCO, short-read mapping, Genome Continuity Inspector (GCI) score, and so on. Minimap2 (v. 2–2.28, RRID:SCR_0185500) [25] and Winnowmap were used to align ONT reads and HiFi reads to the genome assembly, and then the GCI score was calculated by GCI [26].

### Genome annotation

Repeat sequences were identified using a combination of tools to ensure comprehensive detection. RepeatModeler (v. 2.0.4, RRID:SCR_015027) [27] was utilized to predict repeat models based on the genome sequence, while LTR_FINDER (v. 1.07, RRID:SCR_015247) [28] identified long terminal repeat (LTR) sequences. The results from LTR_FINDER were processed with LTR_retriever (v. 2.9.0, RRID:SCR_017623) [29] to eliminate redundancy and construct a *de novo* repeat library. This library was merged with the RepBase database (v. 20181026, RRID:SCR_021169) and analyzed using RepeatMasker (v. 4.0.9, RRID:SCR_012954) [30] to predict repeat sequences. Additionally, RepeatProteinMask was employed to identify TE_protein-type repeats, further enhancing the accuracy and breadth of repeat annotation.

Gene structure prediction utilizes a combination of transcriptome-based, homology-based, and *de novo* approaches. Transcriptome-based prediction reconstructed transcripts using stringtie (v. 2.1.4, RRID:SCR_016323) [31], and coding regions were identified with TransDecoder (v. 5.1.0, RRID:SCR_017647). For homology-based prediction, protein sequences from related species were aligned to the genome using tblastn (v. 2.7.1, RRID:SCR_011822), and transcripts and coding regions were refined using Exonerate (v. 2.4.0, RRID:SCR_016088). *De novo* prediction was performed on repeat-masked genomes using Augustus (v. 3.3.2, RRID:SCR_008417) [32] and Genscan (v. 1.0, RRID:SCR_013362) [33]. The predictions from these methods were integrated with MAKER (v. 2.31.10, RRID:SCR_005309) [34]. To evaluate the completeness of genome annotations, BUSCO (v. 5.2.2, RRID:SCR_015008) was employed.

Protein sequences were aligned to databases such as Uniprot, NR, and the KEGG pathway database using diamond blastp (v. 2.0.11.149) [35]. Functional and pathway information was refined using KOBAS (v. 3.0, RRID:SCR_006350) with KEGG PATHWAY annotations [36, 37]. Gene Ontology (GO) terms were derived through mappings from Uniprot. To identify conserved motifs, protein domains, and structural features, HmmScan (v. 3.3.2) [38] was used with a threshold parameter of -E 0.01. Structural RNAs were predicted with specialized tools: transfer RNAs (tRNAs) were identified using tRNAscan-SE (v. 1.23, RRID:SCR_008637) [39], ribosomal RNA (rRNA) sequences were detected using rRNA databases, and noncoding RNAs (ncRNAs) were annotated with INFERNAL (v. 1.1.2, RRID:SCR_011809) [40] based on the Rfam database. This multifaceted approach provided a comprehensive framework for understanding genome functionality and structure.

### Identifications of centromeres

TRF (Tandem Repeat Finder) (v4.09.1, RRID:SCR_022193) [41] was utilized to search tandem repeats in *de novo* mode and identify locations and monomers of centromeres by BSLtool (v.1.0). The extracted monomer sequence was used as a library with RepeatMasker to rescan the genome. Bedtools (v. 2.30.0, RRID:SCR_006646) [42] was used to intersect the centromeric regions, and these centromeric monomers were visualized and validated by StainedGlass (v. 0.6) [43].

### Gene family identification, phylogenetic inference, and divergence time estimation

Gene family clustering was performed using the OrthoFinder software (v. 2.3.1, RRID:SCR_017118) [44]. In addition to the genes of *C. idella* annotated in this study, protein domains were identified for genes from the following species: *Carassius auratus, Cyprinus carpio, Chanodichthys erythropterus, Carassius gibelio, Danio rerio, Megalobrama amblycephala, Ancherythroculter, Aristichthys nobilis, Mylopharyngodon piceus, Hypophthalmichthys molitrix*, and *Triplophysa tibetana*. Species-specific gene families, referred to as unique gene families, were identified and analyzed for functional enrichment using the clusterProfiler package to perform GO and Kyoto Encyclopedia of Genes and Genomes (KEGG) enrichment analyses [45].

Multiple sequence alignments of protein sequences for each single-copy gene family were performed using MUSCLE (v. 3.8.31, RRID:SCR_011812) [46]. The resulting alignments were concatenated into a supergene dataset, which was used to construct a maximum likelihood (ML) phylogenetic tree using RAxML (v. 8.2.10, RRID:SCR_006086) with the model PROTGAMMAWAG [47]. Phylogenetic trees were generated for *C. idella* and 11 other species (*A. nigrocauda, C. auratus, C. carpio, C. erythropterus, C. gibelio, D. rerio, M. amblycephala, A. nobilis, M. piceus, H. molitrix*, and *T. tibetana*) based on shared single-copy genes. The species tree was rooted using *T. tibetana* and served as input for the MCMCTree program in PAML (RRID:SCR_014932) to construct an ultrametric tree [48]. Secondary calibration points were based on the divergence time between *A. nigrocauda* and *D. rerio* (41.7–68.9 million years ago), as derived from the TimeTree database.

### Positive selection analysis

The single-copy orthologous genes identified between *C. carpio* and *A. nobilis* were used for positive selection analysis using WGDI (v. 0.74) [49]. Protein sequences for these genes were aligned using MUSCLE (v. 3.8.1551, RRID:SCR_011812) [46], and the Ka/Ks values were calculated using the yn00 module of WGDI. A Ka/Ks ratio >1 indicates significant positive selection. For these genes, GO and KEGG enrichment analyses were also performed using the clusterProfiler package (v. 4.0, RRID:SCR_016884) [45].

## Results

### A T2T gapless reference genome for *C. idella*

**Figure 1:**
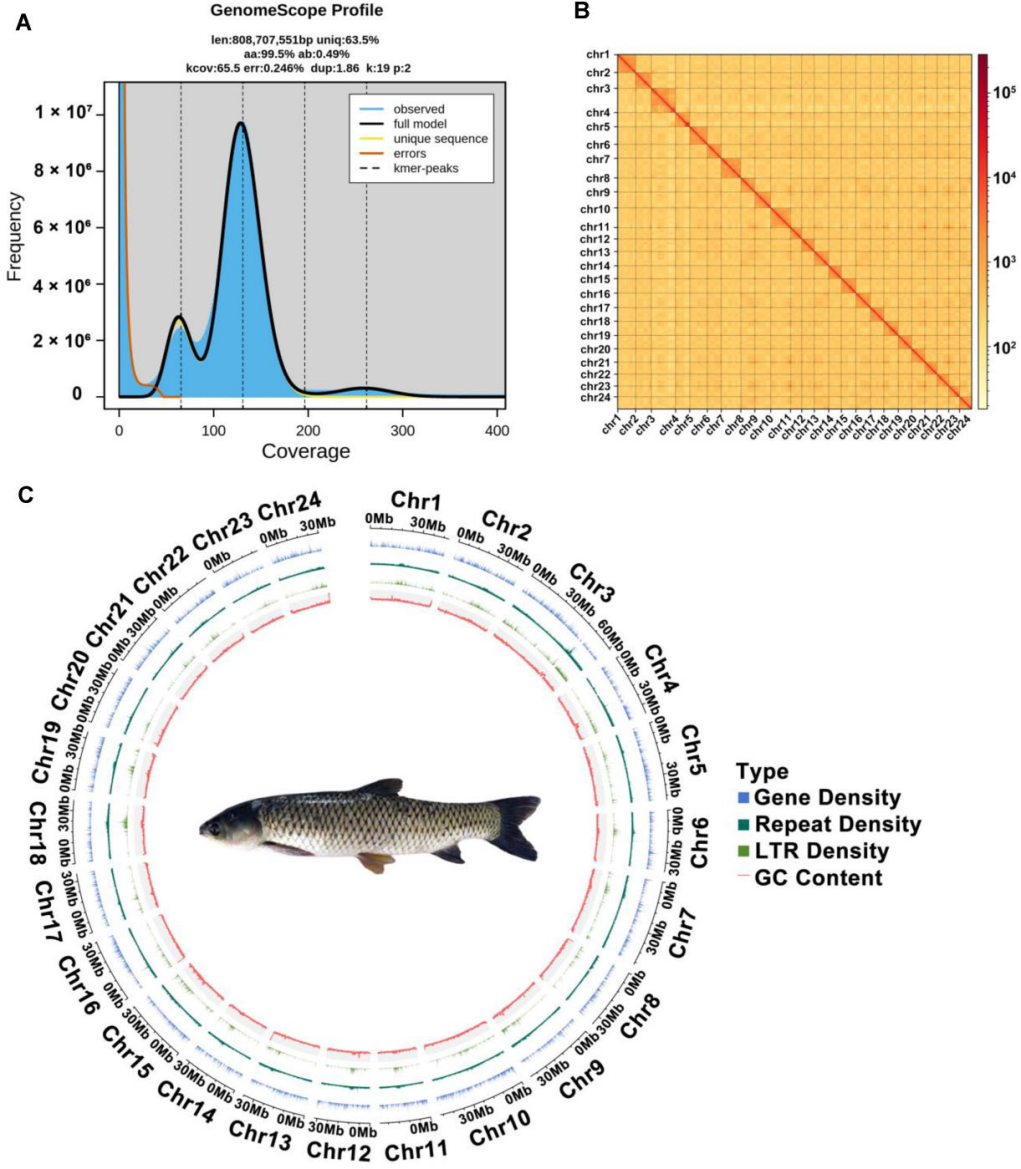
Genome description of *C. idella*. (A) GenomeScope estimation of genome size and heterogeneity using a *k*-mer of 19. (B) Hi-C interaction map. (C) A Circos plot of the assembled pseudochromosomes. Densities were calculated in 100-kb windows.

Various high-coverage sequencing reads were used to develop a gapless genome assembly for *C. idella*. We generated 58.86 Gb (~73×, N50 = 17.4 kb) PacBio HiFi long reads, 73.20 Gb (~91×, N50 = 100.9 kb) ONT ultra-long reads, and 149.61 Gb (~185×) Hi-C sequencing reads, along with 129.10 Gb paired-end reads (159×) (Supplementary Table S1). The genome size was estimated to be 808 Mb with a heterozygosity rate of 0.49% by 19 *k*-mer analysis (Fig. 1A). These various types of sequencing reads were assembled and integrated by 4 strategies using various computational tools, including Hifiasm (HiFi, ONT ultra-long, and Hi-C), verkko (HiFi, ONT ultra-long, and Hi-C), Hifiasm (HiFi and Hi-C), and NextDenovo (ONT ultra-long) [50, 51]. After evaluating these different assemblies, the best assembly, which utilized Hifiasm in combination with HiFi, ONT ultra-long reads, and Hi-C data, was selected as the backbone of the T2T assembly, and the remaining genome assembles were used to fill in gaps or patch telomeres (Supplementary Table S2). The optimal contig assembly spanned 893 Mb with a contig N50 of 35.87 Mb, encompassing 44 telomeres (defined as >200 copies of telomeric repeat units, CCCATTT/TTTAGGG) located at 1 or both ends of 24 contigs. Of these, 18 contigs were classified as T2T, meaning they featured complete, gap-free sequences extending from one telomeric end to the other. We used the ALLHi-C to generate chromosomal interaction maps with Hi-C reads, which demonstrated all 24 chromosomes were gap free. After we added the ribosomal DNA (rDNA) arrays and performed telomere patching, the final T2T gapless assembly of the *C. idella* genome (CyT2T) was 890,918,310 bp on 24 gap-free chromosomes with 48 telomeres. The final chromosome ID and orientation of CyT2T were adjusted in accordance with published version (GCF_019924925.1_HZGC01).

We further performed extensive validations to ensure the accuracy and completeness of the CyT2T assembly in multiple ways. First, Hi-C chromatin interaction maps showed strong consistency across all chromosomes, confirming their accurate arrangement and orientation (Fig. 1B). To estimate the base accuracy, short reads and HiFi reads were mapped to the CyT2T genome, with a mapping rate of 99.88% and 100.00%, respectively. Finally, we evaluated the genomic completeness by BUSCO at 99.1% (3,607 of 3,640 in actinopterygii_odb10) and the Merqury-estimated quality value using short reads at ~49.37 (Supplementary Table S3). The T2T genome achieved an overall GCI score of over 99.99%, with most chromosomes reaching 100%. Collectively, these results show that our final version of the CyT2T gap-free genome has the highest reliability and quality.

### Obtainment of 2 haploid genomes

**Figure 2:**
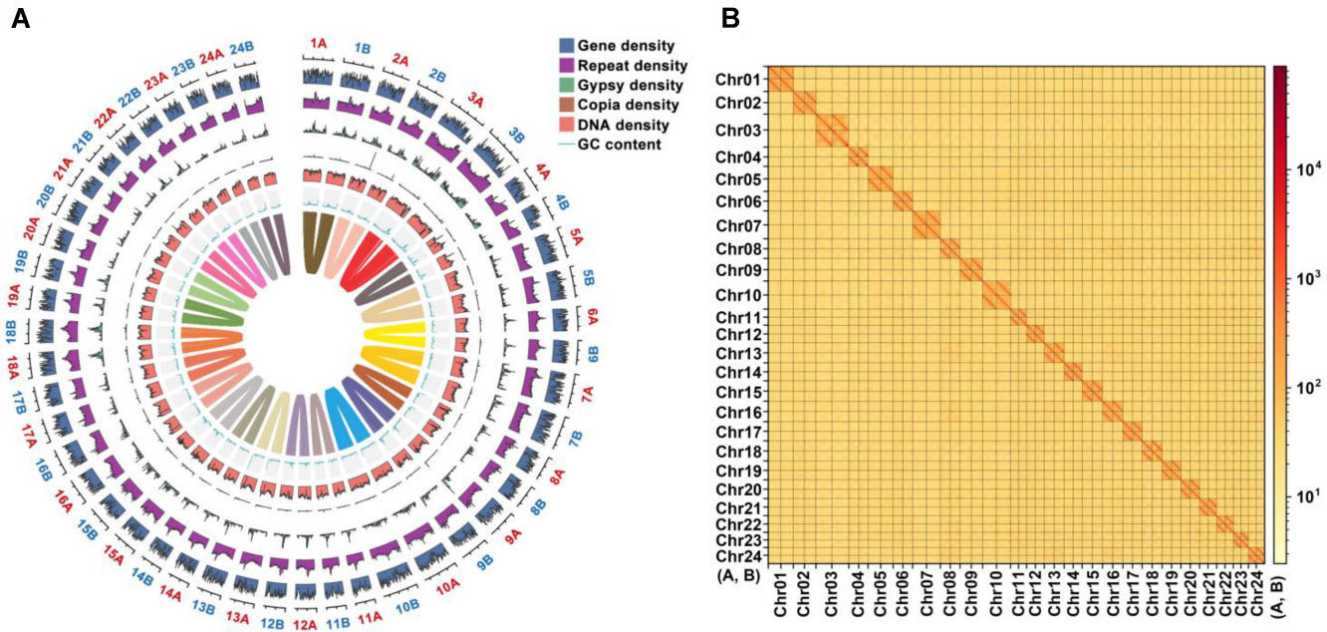
Haploid genomes A and B of *C. idella*. (A) Circos plot of haploid genomes A and B. (I–VII) From outermost to innermost, concentric circles show chromosomes (I), GC content (II), gene density (III), LTR/Gypsy density (IV), LTR/Copia density (V), DNA transposon density (VI), and syntenic regions >100 kb between the A and B haplotype genomes (VII). (B) Hi-C interaction map of haploid genomes A and B.

The haplotype-resolved assembly process successfully produced 2 distinct haploid genomes, designated HapA and HapB, each comprising a complete, gap-free set of chromosomes (Fig. 2 and Supplementary Table S4). To evaluate their quality, we analyzed the assemblies using a combination of second-generation (next-generation sequencing, NGS) and third-generation (long-read) sequencing data. This assessment yielded mapping rates and genome coverage exceeding 99% for both haploid genomes (Supplementary Table S5). High mapping rates indicate precise sequence reconstruction, while near-complete coverage confirms the absence of significant gaps or unassembled regions. Additionally, the assemblies achieved an exceptional quality value of ≥50, the highest among comparable haplotype-resolved genomes (Supplementary Table S6). A quality value of 50 corresponds to an error rate of ≤0.001%, equivalent to no more than 1 base call error per 100,000 base pairs. This unparalleled accuracy highlights the superior quality of HapA and HapB, positioning them as a new benchmark for haplotype-resolved genome assemblies.

To further validate the assembly accuracy, we employed the GCI tool, a quantitative measure of assembly quality based on multiple alignment algorithms. The GCI analysis revealed exceptional assembly continuity for both HapA and HapB. For each chromosome in both haploid genomes, the observed N50 values closely matched the expected N50 values, resulting in GCI scores consistently exceeding 99.99% and often reaching 100% (Supplementary Table S7). These high GCI scores, coupled with the previously mentioned high mapping rates, genome coverage, and quality values, provide compelling evidence for the accuracy and completeness of our haplotype-resolved assemblies.

### Genome annotation

Using an integrated gene annotation pipeline that combined 3 complementary approaches—*de novo* prediction, homologous gene prediction, and RNA sequencing (RNA-seq)–based prediction—we employed 4 specialized tools: Genscan, AUGUSTUS, Exonerate, and TransDecoder. This comprehensive strategy identified 27,446 protein-coding genes. The BUSCO assessment revealed a completeness score of 97.5% for the gene set, comprising 96.2% single-copy genes and 1.3% duplicated genes (Fig. 1C). Functional annotation of these genes was highly successful, with 93.04% of them mapped to entries in 6 major databases: UniProt, Pfam, GO, KEGG, KOG (Eukaryotic Orthologous Groups), and NR (Non-Redundant Protein Database) (Supplementary Tables S8–S9). The protein-coding genes exhibited an average coding sequence length of 1,630 bp and an average of 9.51 exons per gene (Supplementary Table S8). In addition to protein-coding genes, our analysis identified 476.78 Mb of repetitive sequences, which constitute 53.52% of the genome (Supplementary Table S10). Among these, class II transposable elements (TEs), specifically DNA transposons, were the most abundant, accounting for 29.86% of the genome with a total length of 266,062,988 bp. Within class I TEs (retrotransposons), short interspersed nuclear elements (SINEs) were the second-largest contributor, representing 4.87% of the genome and spanning 43.36 Mb (Supplementary Fig. S1). Furthermore, we predicted various types of ncRNA sequences, which are critical for regulatory functions. In total, we identified 2,964 microRNAs (miRNAs), 9,322 rRNAs, 10,097 tRNAs, and 1,734 small nuclear RNAs (snRNAs), collectively covering approximately 2.54 Mb of the genome (Supplementary Table S11).

To ensure the robustness of our assembly, we extended the annotation and prediction analyses to both haplotype genomes, HapA and HapB. We assessed repetitive sequences, gene numbers, BUSCO completeness, and ncRNA content for each haplotype. The high degree of similarity observed between HapA and HapB across these metrics underscores the consistency and accuracy of our haplotype-resolved genome assemblies (Supplementary Table S12).

### Correction of structural variations in the T2T genome assembly

**Figure 3:**
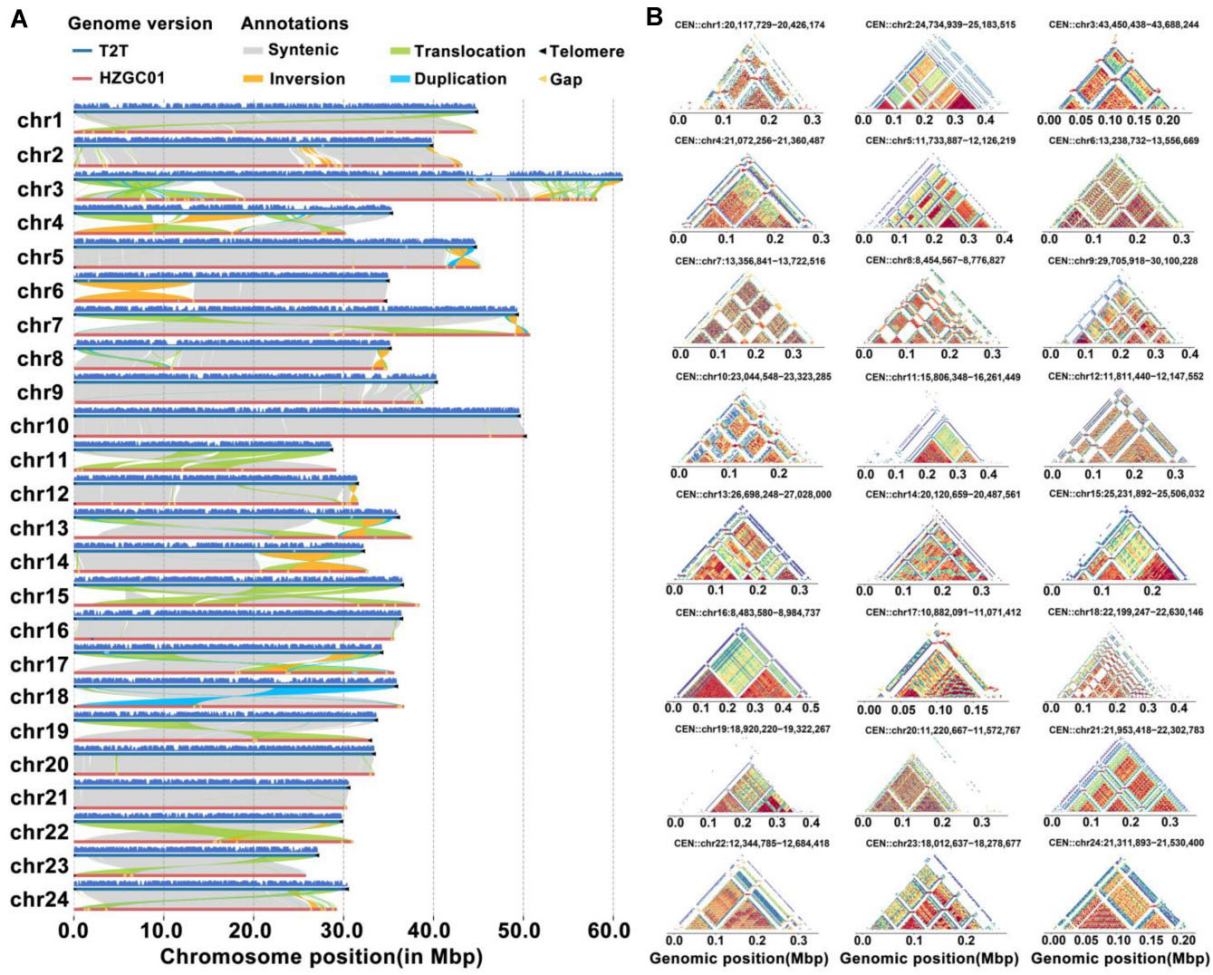
Comparison of the 2 *C. idella* genome versions. (A) Synteny analysis of the 2 *C. idella* genome versions. “T2T” refers to the assembled genome obtained in this study, while “HZGC01” refers to the publicly available genome (NCBI: GCF_019924925.1). (B) StainedGlass sequence identity heatmaps of centromeres for the T2T version.

Compared with the published version (GCF_019924925.1_HZGC01) reference genome, the major improvement in our assembly is that all 150 gaps are filled (Supplementary Table S13). We performed a chromosomal synteny analysis between the assembled *C. idella* genome and *D. rerio* genome, confirming a strong chromosomal synteny relationship between them (Supplementary Fig. S2). Meanwhile, we identified and corrected 38 genomic variations, which span 4 types of structural variations: INV (chromosomal segments inverted relative to their normal orientation), TRANS (chromosomal segments relocated from their original positions to new locations), INVTR (regions that have undergone both inversion and translocation), and INVDP (duplicated chromosomal segments inverted relative to their normal orientation) (Supplementary Table S14). These variations were distributed across 17 chromosomes, covering regions ranging from 1.02 to 13.61 Mb (Fig. 3A). To ensure the accuracy of these corrected regions, we further examined the read coverage within these variant regions (Supplementary  Fig. S3). Additionally, the completed T2T version of the genome also improved the existing publicly available *C. idella* genome by filling 150 gap regions. This includes the completion of gaps, which involve 108 genes across 12 chromosomes (Supplementary Table S15). These results demonstrate the significant improvements made in the T2T version of the *C. idella* genome compared to the currently available version.

### Analysis of centromeric monomers

The centromeres in the genome were identified and characterized based on their genomic positions, lengths, and monomer compositions. The 24 chromosomes exhibited centromeres with lengths ranging from 189,322 bp (chr17) to 501,158 bp (chr16), with an average length of approximately 340,000 bp (Fig. 3B and Supplementary Table S16). Detailed annotations revealed distinct patterns of centromeric organization across chromosomes. For instance, chr2 had a centromere spanning 448,577 bp (24,734,939–25,183,515), while chr16 contained the largest centromere, spanning 501,158 bp (8,483,580–8,984,737). Conversely, chr17 had the smallest centromere at 189,322 bp (10,882,091–11,071,412). The findings provide a comprehensive understanding of the centromeric architecture, revealing variation in centromere length and organization among chromosomes, highlighting their unique structural characteristics.

### Expansion and contraction of gene families and phylogenetic inference

**Figure 4:**
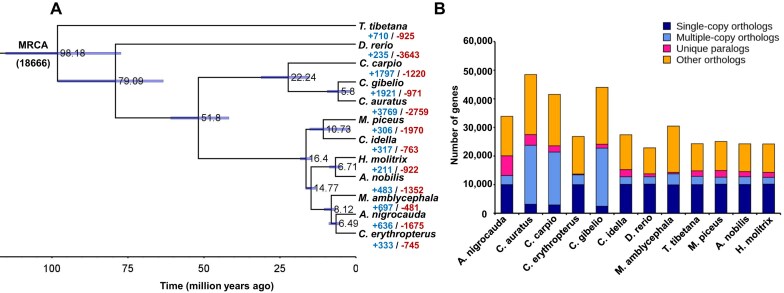
Comparative genomic analysis of *C. idella* and its related species. (A) Phylogenetic tree representing the number of gene families that have expanded or contracted among 12 species. The number at the root (18,666) denotes the total number of gene families predicted in the most recent common ancestor (MRCA). The estimated divergence time (in millions of years) is shown beside the branch nodes in black. The scale on the x-axis shows the estimated divergence time for nodes. “+” indicates that gene families expanded, and “−” indicates that gene families contracted. (B) The prediction of single-copy and multicopy gene families across 12 species.

We investigated the expansion and contraction of gene families during the evolution of Cyprinidae. Our focus was on the impact of polyploidization events in Cyprinidae, which corresponded to 9,740 expanded and 473 contracted gene families in the Cyprininae subfamily (including *Cyprinus carpio, Carassius auratus*, and *Carassius gibelio*) (Fig. 4A, Supplementary Tables S17–S18). Additionally, we examined 4 major Asian domestic carps: grass carp (*C. idella*), black carp (*Mylopharyngodon piceus*), bighead carp (*Aristichthys nobilis*), and silver carp (*Hypophthalmichthys molitrix*). We identified 84 uniquely expanded and 1,612 contracted gene families in the Hypophthalmichthyinae subfamily for *A. nobilis* and *H. molitrix*, as well as 23 uniquely expanded and 541 contracted gene families in the Leuciscidae subfamily for *M. piceus* and *C. idella*. Notably, grass carp, a herbivorous fish, exhibited 317 uniquely expanded and 763 contracted gene families. Additionally, we classified the gene family clusters into 4 categories: single-copy orthologs, multiple-copy orthologs, species-specific genes (unique paralogs), and other orthologs. In *C. idella*, we identified 18,686 gene families, including 10,112 single-copy orthologs, 2,639 multiple-copy orthologs, and 2,493 species-specific genes (Fig. 4B).

Using single-copy orthologous genes, we constructed a phylogenetic tree, which revealed that *D. rerio* diverged from other Cyprinidae species approximately 79.1 million years ago (MYA). Furthermore, based on the phylogenetic tree and fossil calibration, we estimated that the divergence between *C. idella* and *M. piceus* occurred around 10.7 MYA, while the divergence between *A. nobilis* and *H. molitrix*, both members of the “Four Major Chinese Carps,” occurred approximately 6.7 MYA (Fig. 4A).

GO and KEGG enrichment analyses revealed that expanded gene families in grass carp are associated with sensory and food-related activities, including olfactory receptor activity (GO: 0004984), G-protein coupled receptor activity (GO: 0004930), and the G-protein coupled receptor signaling pathway (GO: 0007186) (Fig. 4A, Supplementary Table S19, and Supplementary Fig. S4). In contrast, contracted gene families are enriched in digestion-related functions, such as serine-type endopeptidase activity (GO: 0004252) and metalloendopeptidase activity (GO: 0004222) (Fig. 4A, Supplementary Table S20, and Supplementary  Fig. S4). These findings suggest a potential association between the expansion and contraction of gene families and the dietary evolution of grass carp.

### Ka/Ks analysis

We calculated the Ka/Ks values between *C. carpio* and *A. nobilis* and identified 263 genes as positively selected genes (PSGs) (Ka/Ks > 1) (Supplementary Table S21). GO analysis of these PSGs revealed that 3 genes were associated with the adaptive immune response (GO: 0002250, *P* < 0.001), 4 genes are linked to chemokine activity (GO: 0008009, *P* = 0.002), and 2 genes are related to G-protein beta-subunit binding (GO: 0031681, *P* = 0.005) (Supplementary Table S22).

### Reuse potential

The T2T genome of grass carp (*C. idella*), a large herbivorous freshwater fish from the Cyprinidae family, represents a groundbreaking and highly versatile genomic resource with immense potential for advancing research and practical applications. As the most economically significant freshwater aquaculture species in China and globally, grass carp is a cornerstone of food production and is revered as one of the Four Great Domestic Fishes. However, prior genome assemblies of this species have been hampered by gaps and incompleteness, limiting progress in molecular research and genetic enhancement. This T2T gap-free assembly, spanning 890,918,310 bp across 24 chromosomes, achieves unprecedented completeness and quality, providing an exceptional foundation for exploring the species’ genomic landscape and supporting its genetic improvement.

This T2T assembly encompasses 27,446 protein-coding genes, with 93.04% annotated using multiple databases, alongside the characterization of 48 telomeres and 24 centromeres. The gap-free reference genome enables detailed investigation of centromere structures, revealing conserved centromere-specific satellite motifs unique to grass carp. This resource empowers researchers to probe evolutionary dynamics and functional variations, potentially identifying genes linked to traits like growth rate or disease resistance—crucial for aquaculture breeding programs. By serving as a comprehensive reference, this T2T genome paves the way for future studies, such as haplotype-resolved analyses or the development of a Cyprinidae pan-genome, to further illuminate adaptive traits and species divergence.

## Discussion

Grass carp, as the most economically important fish species in both China and worldwide, holds significant potential for advancements in genomic breeding technologies, such as whole-genome selection and other molecular breeding approaches [1, 2]. In this study, we present the first T2T genome assembly of grass carp. To achieve this, we employed an integrated sequencing approach, utilizing high-depth DNB-T7 short reads, PacBio HiFi long reads, and Hi-C technology reads, alongside ONT ultra-long reads. This diverse approach allows for the generation of a high-quality, chromosome-scale reference genome, resolving a number of previously reported assembly errors.

Compared to the previously published chromosome-level genome [14], our assembly corrects 38 assembly errors, fills 150 genomic gaps, and completes the assembly of previously unresolved telomeric and centromeric regions. This refinement of the grass carp genome offers a more comprehensive and accurate representation of its genomic architecture. The improved genome assembly provides a robust resource for more accurate surveys of germplasm resources across different regions, population genetic analyses, and breeding studies in grass carp, potentially accelerating the development of genetically improved strains for aquaculture.

Beyond its utility in breeding and genomic research, our study also contributes to a deeper understanding of the evolutionary mechanisms shaping the Cyprinidae family, particularly in terms of gene family expansions and contractions. These analyses revealed distinct patterns in gene family dynamics, which are likely linked to the unique ecological niche and dietary preferences of grass carp. For example, we identified specific gene family expansions related to sensory perception and food-related activities, such as olfactory receptors and G-protein coupled receptors. These findings suggest that grass carp, as a herbivorous species, may have evolved specialized molecular mechanisms to adapt to its unique feeding behavior.

This comprehensive genome also provides a solid foundation for further comparative genomics studies in the Cyprinidae family and offers new opportunities for exploring the evolutionary relationships between grass carp and other species. With more detailed genomic data, we can now better understand how grass carp has evolved to thrive in diverse aquatic environments and how its genome may have adapted to the specific demands of herbivorous feeding.

Overall, this study not only enriches our understanding of the grass carp’s biology and evolution but also contributes to the broader field of teleost fish genomics. By leveraging the full potential of this high-quality genome, we can further explore the genetic underpinnings of key traits in grass carp, facilitating improvements in breeding programs and providing valuable insights into the broader evolutionary dynamics of freshwater fish.

## Supplementary Material

giaf059_Supplemental_Files

giaf059_Authors_Response_To_Reviewer_Comments_original_submission

giaf059_GIGA-D-25-00078_original_submission

giaf059_GIGA-D-25-00078_Revision_1

giaf059_Reviewer_1_Report_original_submission Kun Wang -- 4/3/2025

giaf059_Reviewer_2_Report_original_submission Li Ren -- 4/6/2025

## Data Availability

The genomic sequence and RNA-seq data of *C. idella* generated by this study were deposited in NCBI under accession number PRJNA1251791 and NGDC (National Genomics Data Center) database under accession number PRJCA036158. The assembled genome sequences and annotation information have been submitted in NGDC under accession number PRJCA036158. All additional supporting data are available in the *GigaScience* repository, GigaDB [52].

## Additional Files

**Supplementary Figure S1**. Chromosome synteny analysis of zebrafish and grass carp.

**Supplementary Figure S2**. Distribution of transposable element (TE) for *C. idella* genome.

**Supplementary Figure S3**. Read coverage for the variant regions between the T2T and HZGC01 versions of the *C. idella* genome.

**Supplementary Table S1**. Overview of data utilized for genome assembly of *Ctenopharyngodon idella*.

**Supplementary Table S2**. Evaluation of contig-level genome assemblies generated by different assembly strategies.

**Supplementary Table S3**. BUSCO assessment for the *Ctenopharyngodon idella* genome assembly.

**Supplementary Table S4**. Chromosome information of the haploid genome.

**Supplementary Table S5**. Alignment statistics of haplotype genomes.

**Supplementary Table S6**. Highest quality value (QV) assessment of haplotype genomes.

**Supplementary Table S7**. GCI analysis results for HapA and HapB chromosomes.

**Supplementary Table S8**. Gene prediction statistics.

**Supplementary Table S9**. Gene annotation of the assembled genome.

**Supplementary Table S10**. Summary of repeat contents.

**Supplementary Table S11**. The summary of predicted noncoding RNA in grass carp.

**Supplementary Table S12**. BUSCO assessment and gene annotation of haploid genomes A and B.

**Supplementary Table S13**. Overview of genome assembly.

**Supplementary Table S14**. Comparative analysis of the assembled *Ctenopharyngodon idella* genome and the publicly available version (HZGC).

**Supplementary Table S15**. Completed gap information for the assembled *Ctenopharyngodon idella* genome, in comparison to the publicly available version (HZGC).

**Supplementary Table S16**. Summary of centromere prediction.

**Supplementary Table S17**. Information of 11 downloaded genomes.

**Supplementary Table S18**. Summary of gene family clustering results.

**Supplementary Table S19**. GO enrichment of expanded gene families in *C. idella*.

**Supplementary Table S20**. GO enrichment of contracted gene families in *C. idella*.

**Supplementary Table S21**. Positively selected genes (PSGs) between *C. carpio* and *A. nobilis*.

**Supplementary Table S22**. GO annotation of PSGs between *C. carpio* and *A. nobilis*.

## Abbreviations

BUSCO: Benchmarking Universal Single-Copy Orthologs; GO: Gene Ontology; HiFi: high fidelity; INV: chromosomal segments inverted relative to their normal orientation; INVDP: duplicated chromosomal segments inverted relative to their normal orientation; INVTR: regions that have undergone both inversion and translocation; KEGG: Kyoto Encyclopedia of Genes and Genomes; LTR: identified long terminal repeat; miRNA: microRNA; ML: maximum likelihood; MYA: million years ago; ncRNA: noncoding RNA; ONT: Oxford Nanopore Technologies; PSG: positively selected gene; rRNA: ribosomal RNA; SINE: short interspersed nuclear element; snRNA: small nuclear RNA; T2T: telomere-to-telomere; TE: transposable element; TRANS: chromosomal segments relocated from their original positions to new locations; TRF: Tandem Repeat Finder; tRNA: transfer RNA.
