# Correction to: The telomere-to-telomere gapless genome of grass carp provides insights for genetic improvement

**DOI:** 10.1093/gigascience/giaf088

**Published:** 2025-07-15

This is a correction to: Fei Liu, Yuan Li, Guishuang Wang, Dong Zhang, Xinlan Yang, Chaowei Zhou, Rongzhu Zhou, Haiping Liu, The telomere-to-telomere gapless genome of grass carp provides insights for genetic improvement, *GigaScience*, Volume 14, 2025, giaf059, https://doi.org/10.1093/gigascience/giaf059.

Several corrections have been made to the originally published version of this manuscript.

In Methods, in the following sentence in the section entitled “Sample collection and sequencing,” “A wild female *C. idella*” should have read “A wild male *C. idella*“:

A wild female *C. idella* collected from Hunan Fisheries Science Institute, Changsha, Hunan, China, was used in construction of the reference genome.

The corrected sentence reads:

A wild male *C. idella* collected from Hunan Fisheries Science Institute, Changsha, Hunan, China, was used in construction of the reference genome.

In Methods, in the following sentence in the section entitled “Gene family identification, phylogenetic inference, and divergence time estimation,” “*A. nigrocauda*” should have read “*T. tibetana*” in the following sentence:

The species tree was rooted using *A. nigrocauda* and served as input for the MCMCTree program in PAML (RRID:SCR_014932) to construct an ultrametric tree [48].

The corrected sentence reads:

The species tree was rooted using *T. tibetana* and served as input for the MCMCTree program in PAML (RRID:SCR_014932) to construct an ultrametric tree [48].

In Figure 4, the Latin names of the two species were mistakenly swapped. The original Figure has been replaced with the corrected version.

