# Supplementary material for: The telomere-to-telomere gapless genome of grass carp provides insights for genetic improvement
Source: Gigascience. 2025 Jun 18;14:giaf059. doi: 10.1093/gigascience/giaf059 (PMC12204074; doi:10.1093/gigascience/giaf059)
Supplement: giaf059_Supplemental_Files [file giaf059_supplemental_files.zip › 2025.06.17 Supplementary Figures.docx]

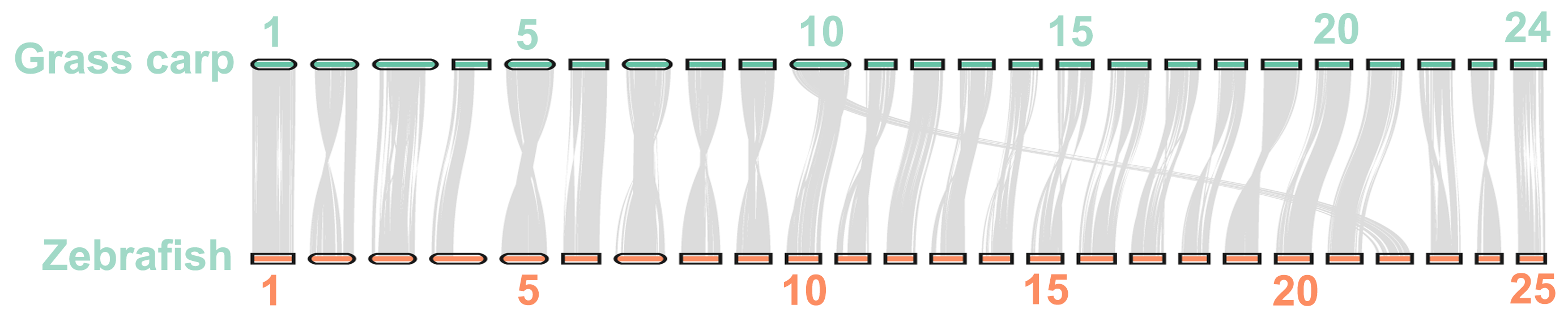


**Figure S1. Chromosome synteny analysis of zebrafish and grass carp.**


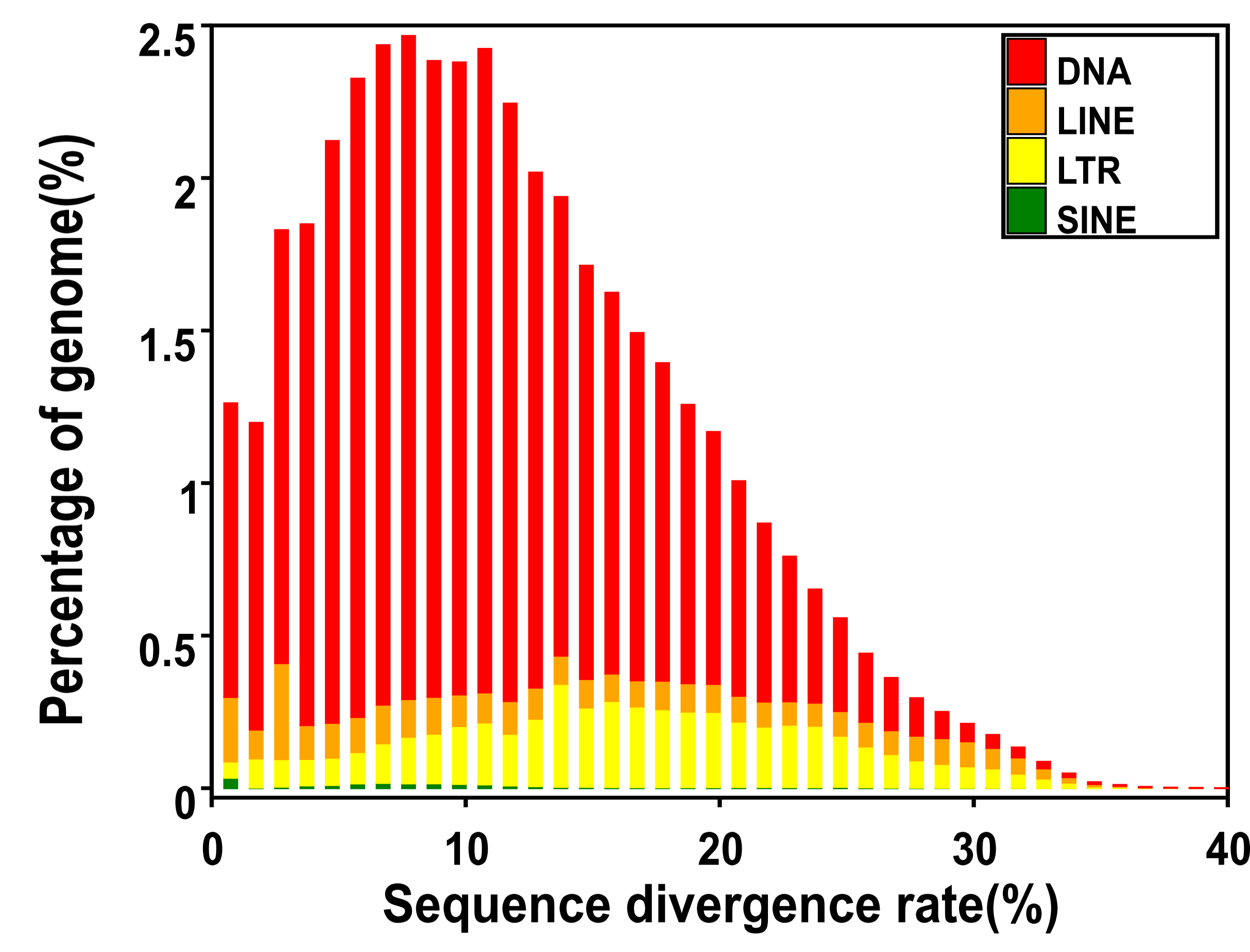


**Figure S2. Distribution of transposable element (TE) for *C. idella* genome*.***


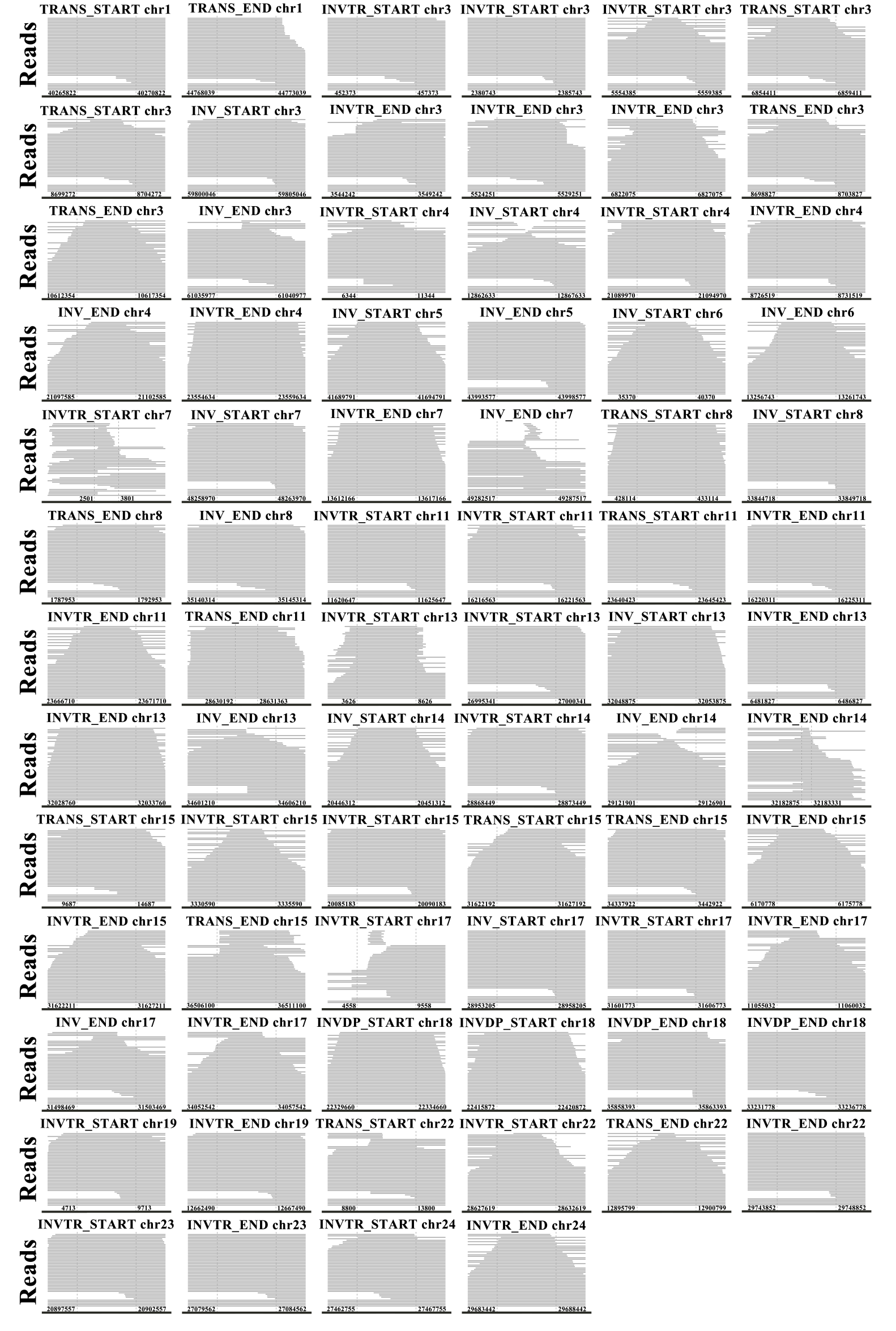


**Figure S3. Read coverage for the variant regions between the T2T and HZGC01 versions of the *C. idella* genome.**
