# Supplementary material for: The telomere-to-telomere gapless genome of grass carp provides insights for genetic improvement
Source: Gigascience. 2025 Jun 18;14:giaf059. doi: 10.1093/gigascience/giaf059 (PMC12204074; doi:10.1093/gigascience/giaf059)
Supplement: giaf059_GIGA-D-25-00078_Revision_1 [file giaf059_giga-d-25-00078_revision_1.pdf]

GigaScience

The telomere-to-telomere gapless genome of grass carp provide insights for genetic improvement

--Manuscript Draft--

**Manuscript Number:**	GIGA-D-25-00078R1
**Full Title:**	The telomere-to-telomere gapless genome of grass carp provide insights for genetic improvement
**Article Type:**	Data Note
**Funding Information:**	Guangzhou Key research and development Program (2024B03J1082) — Reseacher Fei Liu<br>NSFC Joint Fund Priority Support Program (U23A20249) — Reseacher Fei Liu
**Abstract:**	Background<br>The grass carp (Ctenopharyngodon idella) is a large herbivorous freshwater fish belonging to the Cyprinidae family. It is widely cultivated as a food source in China and is renowned as one of the Four Great Domestic Fishes. Despite its economic importance, the published genome assemblies of grass carp remain incomplete due to gaps, thereby hindering molecular research and genetic improvement.<br><br>Results<br>In this study, we report the assembly of a telomere-to-telomere (T2T) gap-free genome of the grass carp with total length of 890,918,310 bp for 24 chromosomes without gaps, representing the highest completeness and assembly quality to date.<br>Our assembly contains 27,446 protein-coding genes and 93.04% of all were annotated with multiple databases, with 48 telomeres and 24 centromeres characterized. Gap-free reference genome enable us study the structure of centromeres and identify conserved centromere-specific satellite motifs for grass carp. Furthermore, we identified 108 gene-related gaps across 12 chromosomes and 38 structural variations across 17 chromosomes in this T2T assembly.<br><br>Conclusions<br>The validated gap-free genome for provides invaluable resource for future genomic studies grass carp, offering new insights into its genetic architecture and evolutionary dynamics.
**Corresponding Author:**	Rongzhu Zhou<br>National Animal Husbandry Services<br>Beijing, CHINA
**Corresponding Author Secondary Information:**	
**Corresponding Author's Institution:**	National Animal Husbandry Services
**Corresponding Author's Secondary Institution:**	
**First Author:**	Fei Liu
**First Author Secondary Information:**	
**Order of Authors:**	Fei Liu<br>Yuan Li<br>Guishuang Wang<br>Dong Zhang<br>Xinlan Yang

Powered by Editorial Manager® and ProduXion Manager® from Aries Systems Corporation

	Chaowei Zhou
	Haiping Liu
	Rongzhu Zhou
**Order of Authors Secondary Information:**	
**Response to Reviewers:**	Dear Dr. Hongfang Zhang,<br>Please find the revised manuscript entitled "The telomere-to-telomere gapless genome of grass carp provide insights for genetic improvement" (GIGA-D-25-00078), which we would like to submit for publication as original research in GigaScience.<br>The comments provided by the editors and reviewers have enabled us to further improve the quality of our manuscript. The grammar and formatting errors have been comprehensively corrected throughout the text. We have highlighted the revised sections in yellow. Detailed explanations and our point-by-point responses to each of the comments are provided in the following pages.<br><br>We are looking forward to hearing from you at your earliest convenience.<br><br>Sincerely!<br><br>Prof. Rongzhu Zhou<br>National Animal Husbandry Services, Beijing 100125, China<br><br>Responses to the comments of the editor:<br>1. Looking at this the referencing is totally messed up - as we use chronological and numerical, while it's alphabetical in your manuscript. Please redo the referencing so the copy editor doesn't mess this up. Please ensure that the reference style is followed precisely and consistently. You can refer to the author guidelines https://academic.oup.com/gigascience/pages/data_note for more details.<br>Answer<br>Thank you for your kind comments. As per your suggestion, we have revised the reference formatting accordingly.<br><br>2. In the methods section it would be useful if you add RRID details to some of the software tools and sequencers. These needed to be listed after resources in brackets. If you are citing papers for these resources, the RRID do not replace these, and both should be included. This can be included in the methods section of the paper similar to the RRIDs included here:<br>DNBSEQ-T7 (RRID:SCR_017981); PacBio Sequel II System (RRID:SCR_017990); PLINK (RRID:SCR_001757)<br>Answer<br>Thank you for your kind comments. We have added the RRID details for the software tools and sequencing platforms used in the Methods section of the revised manuscript.<br><br>Responses to the comments of the reviewer 1:<br>This manuscript presents the generation and initial characterization of a telomere-to-telomere (T2T), gapless genome assembly for the economically important grass carp, Ctenopharyngodon idella. The authors utilized a state-of-the-art combination of PacBio HiFi, Oxford Nanopore ultra-long reads, Hi-C, and Illumina sequencing, achieving impressive assembly quality metrics (T2T chromosomes, high BUSCO, QV, and GCI scores). The methods are detailed, validation is thorough, and the data availability statement is clear. While the manuscript is generally strong, a few minor points could further improve its clarity and impact.<br>Answer<br>We sincerely appreciate your valuable feedback, which has significantly enhanced the quality and clarity of our work. In the following sections, we have addressed each of your comments with detailed revisions and responses.<br><br>1.The Introduction states that Mastacembelus armatus is the only fish (at the time of writing) with a chromosome-level T2T assembly. While likely true when drafted, the T2T field moves rapidly. Please double-check if any other fish T2T assemblies (chromosome-level) have been published very recently and update the statement if necessary for accuracy at the time of publication.<br>Answer

Powered by Editorial Manager® and ProduXion Manager® from Aries Systems Corporation

	Yes, we have collected information on fish species that have recently published T2T genome versions, and indeed, we found that several fish species have been sequenced. We have updated the Introduction section accordingly. The revision is as follows:<br>"Several fish species, including Mastacembelus armatus (Xue et al. 2021), Clarias gariepinus (Nguinkal et al. 2024), Rhinogobio nasutus (Jiang et al. 2025), have also been sequenced to a chromosome-level T2T genome assembly." (Page 3, lines 23-25)<br><br>2.The authors state they used different assembly strategies/tools (Hifiasm, verkko, NextDenovo) and selected the "best assembly" (Hifiasm with HiFi+ONT+Hi-C) as the backbone. The criteria for "best" are not explicitly stated or quantified comparatively.<br>Answer<br>Thank you for the kind comments. We have included a new Table S2 in the latest revised version to provide detailed information on the genome assemblies generated using multiple assembly strategies.<br>Table S2. Evaluation of contig-level genome assemblies generated by different assembly strategies.<br>Versions of contig-level genome assembliesLength (bp)N50 (bp)Quality ValueNumberBUSCODepth (X)<br>Hifiasm (HIFI+HIC)17732547533356109351.38139C:99.1%[S:0.1%,D:99.0%]33.19<br>Hifiasm (HIFI+ONT+HIC)17724388643567773551.2395C:99.1%[S:0.1%,D:99.0%]33.21<br>Verkko (HIFI+HIC)17807398881862625850.67669C:99.1%[S:0.3%,D:98.8%]33.05<br>Verkko (HIFI+ONT+HIC)17774710753556738450.83243C:99.1%[S:0.1%,D:99.0%]33.11<br>*"C" represents complete BUSCOs, "S" indicates complete and single-copy BUSCOs, and "D" indicates complete but duplicated BUSCOs.<br><br>3.Title, 'genome provide insights' should be 'genome provides insights'<br>Answer<br>Thank you for the kind comments. We have revised it.<br><br>4.Page 4: The latin name should be italized. This should be checked across the whole manuscript.<br>Answer<br>We have thoroughly checked and revised the entire manuscript. Thank you for your valuable comments.<br><br>5. Page 7: The line spacing is not unified.<br>Answer<br>We have thoroughly checked and revised the entire manuscript. Thank you for your valuable comments.<br><br>Responses to the comments of the reviewer 2:<br>1.Grass carp (Ctenopharyngodon idella) is the most productive freshwater aquaculture species in China. Each year, a large number of studies are published on its genetic improvement, immunity, and nutrition. The newly reported T2T genome in this study provides a valuable reference for researchers working in these areas, both domestically and internationally. As a data note, this manuscript presents a relatively comprehensive analysis of the newly assembled genome. Compared to previously published grass carp genomes, this version shows notable improvements in quality, particularly in telomeric, centromeric, and other repetitive regions. I recommend acceptance after minor revision.<br>Answer<br>Thank you for the kind comments! In the latter part of this document, we have provided detailed point-by-point responses to the comments raised.<br><br>1.Sample Information: Clarify the sex (male or female) of the grass carp individual used for genome sequencing.<br>Answer<br>We selected a male grass carp as the specimen for genome sequencing. The following statement has been added to the Methods section:<br>"A wild female C. idella collected from Hunan Fisheries Science Institute, Changsha, Hunan, China, was used in construction of the reference genome." (page 4, lines 2-3)

	2.Genome Size Discrepancy: The manuscript reports an estimated genome size of 808 Mb but a final T2T assembly size of ~891 Mb. Explain the difference (e.g., k-mer estimation vs. assembly output).<br>Answer<br>These two values represent different aspects of the genome and should not be confused. The "808 Mb" refers to the initial genome size estimated using k-mer analysis, while the "891 Mb" corresponds to the final size of the completed T2T assembly. This discrepancy highlights that genome size estimates based on k-mer analysis may not fully reflect the actual size of the genome.<br><br>3.T2T Chromosome Definition: The statement "44 telomeres... 18 of which were T2T" is unclear, given grass carp has 24 chromosomes. Elaborate on the criteria for designating chromosomes as T2T, as only 18 are labeled as such despite the T2T (gap-free) implication.<br>Answer<br>To improve clarity, we have revised the sentence as follows:<br>"The optimal contig assembly spanned 893 Mb with a contig N50 of 35.87 Mb, encompassing 44 telomeres (defined as >200 copies of telomeric repeat units, CCCATTT/TTTAGGG) located at one or both ends of 24 contigs. Of these, 18 contigs were classified as telomere-to-telomere (T2T), meaning they featured complete, gap-free sequences extending from one telomeric end to the other." (page 7, lines 12-16)<br><br>4.Terminology Consistency: In the Results section, "CT2T" appears—should this be "CyT2T" as used earlier? Ensure consistent terminology throughout.<br>Answer<br>Thank you for the valuable comments. We have implemented the changes.<br><br>5.Italicize the Latin name Ctenopharyngodon idella in the Methods section under "Sample collection and sequencing" (e.g., "Short-read libraries of C. idella...").<br>Standardize sequencing depth notation in the Results section (e.g., use "~73×," "~91×," "~185×" consistently instead of mixing "73×" and "91×").<br>Fix general formatting errors: remove double spaces, standardize "HiFi" (not "HIFI"), and adjust the section title "Positive Selection Analysis" to "Positive selection analysis" (capitalize only the first word).<br>Add line numbers to the manuscript for easier review.<br>Answer<br>Thank you for the valuable comments. We have revised the errors as described.<br><br>6.Grammatical and Clarity Improvements:<br>Revise "Different and high-coverage sequencing reads were used" to "Various high-coverage sequencing reads were used" for smoother phrasing.<br>Correct "The rest genome assembles were used" to "The remaining genome assemblies were used."<br>Add a subject to "After addition of the rDNA arrays and telomere patching..." (e.g., "After we added the rDNA arrays and performed telomere patching...").<br>Simplify "Hi-C chromatin interaction maps exhibited consistent consistency" (redundant) to "Hi-C chromatin interaction maps showed strong consistency" or similar.<br>Change "three genes are associated" to "were associated" for consistent past tense.<br>Answer<br>Thank you for the valuable comments. We have revised the errors as described.<br><br>Precision and Clarity:<br>The "GCI score of 99.9999%" seems overly precise. Round to 99.99% unless the method justifies this level of detail.<br>Answer<br>Thank you for the valuable comments. We have revised it as below:<br>"The T2T genome achieved an overall GCI score of over 99.99%, with most chromosomes reaching 100%."(page 7, lines 12-16)<br><br>Reference:<br>Jiang C, Du Y, Lou Z, Zhang Y, Wang T. 2025. Telomere-to-telomere reference genome of Rhinogobio nasutus, an endangered endemic fish from the Yellow River. Scientific Data 12(1): 462.

Powered by Editorial Manager® and ProduXion Manager® from Aries Systems Corporation

	Nguinkal JA, Zoclanclounon YA, Brunner RM, Chen Y, Goldammer T. 2024. Haplotype-resolved and near-T2T genome assembly of the African catfish (Clarias gariepinus). Scientific Data 11(1): 1095.<br>Xue L, Gao Y, Wu M, Tian T, Fan H, Huang Y, Huang Z, Li D, Xu L. 2021. Telomere-to-telomere assembly of a fish Y chromosome reveals the origin of a young sex chromosome pair. Genome biology 22(1): 203.
Additional Information:	
Question	Response
Are you submitting this manuscript to a special series or article collection?	No
**Experimental design and statistics**<br><br>Full details of the experimental design and statistical methods used should be given in the Methods section, as detailed in our Minimum Standards Reporting Checklist. Information essential to interpreting the data presented should be made available in the figure legends.<br><br>Have you included all the information requested in your manuscript?	Yes
**Resources**<br><br>A description of all resources used, including antibodies, cell lines, animals and software tools, with enough information to allow them to be uniquely identified, should be included in the Methods section. Authors are strongly encouraged to cite Research Resource Identifiers (RRIDs) for antibodies, model organisms and tools, where possible.<br><br>Have you included the information requested as detailed in our Minimum Standards Reporting Checklist?	Yes
**Availability of data and materials**<br><br>All datasets and code on which the conclusions of the paper rely must be either included in your submission or deposited in publicly available repositories (where available and ethically	Yes

Powered by Editorial Manager® and ProduXion Manager® from Aries Systems Corporation

appropriate), referencing such data using a unique identifier in the references and in the “Availability of Data and Materials” section of your manuscript.<br><br>Have you have met the above requirement as detailed in our Minimum Standards Reporting Checklist?	
GigaScience has policies and guidelines in place for the use of generative AI-writing tools such as ChatGPT. If you have used such writing tools to assist with writing the manuscript this must be declared and cited in the text. Authors should not list AI-writing tools and other AI-assisted technologies as an author or co-author and should acknowledge that they are fully responsible for text generated or refined by AI-writing tools.<p><br><br>A summary of use (particularly in the introduction or among methods) needs to be included at the end of the paper, and the outputs should also be included as a supplementary file hosted in GigaDB or other open repositories. Please read our guidelines for more information. <p><br><br>By submitting to GigaScience, you are aware of the journal's AI-writing tools policy, and if you have declared use of such tools below, you have acknowledged this where appropriate in your manuscript and have made a summary of use and outputs available. <p><br><b>AI-assisted writing tools have been used in the preparation of this manuscript?	No

Manuscript

Click here to access/download;Manuscript;Manuscript-20250430.docx

The telomere-to-telomere gapless genome of grass carp provides insights for genetic improvement

Fei Liu[1, 2, 3]*, Yuan Li[5]*, Guishuang Wang[2], Dong Zhang[1], Xinlan Yang[2], Chaowei Zhou[2,3], Rongzhu Zhou[4]† Haiping Liu[1, 3]†

1. School of Ecology and Environment, Tibet University, Lhasa, Tibet 850000, China;

2. Institute of Aquatic Sciences, Tibet Autonomous Region Academy of Agricultural and Animal Husbandry Sciences, Lhasa, Tibet 850000, China;

3. Integrative Science Center of Germplasm Creation in Western China (CHONGQING) Science City, Key Laboratory of Freshwater Fish Reproduction and Development (Ministry of Education), Key Laboratory of Chongqing Municipality for Aquatic Economic Animal Resources Conservation and Germplasm Creation, College of Fisheries, Southwest University, Chongqing 400715, China;

4. National Animal Husbandry Services, Beijing 100125, China；

5. Wuhan Huabiology Co., Ltd. , Wuhan, Hubei 430000, China

†Corresponding address. Haiping Liu, Integrative Science Center of Germplasm Creation in Western China (CHONGQING) Science City, Key Laboratory of Freshwater Fish Reproduction and Development (Ministry of Education), Key Laboratory of Chongqing Municipality for Aquatic Economic Animal Resources Conservation and Germplasm Creation, College of Fisheries, Southwest University, Chongqing 400715, China. Email:luihappying@163.com

Rongzhu Zhou, National Animal Husbandry Services, Beijing 100125, China. Email:1109904665@qq.com

*These authors contributed equally to this work.

Fei Liu [0009-0004-8000-5596]; Yuan Li [0000-0002-4802-0305]; Guishuang Wang; Dong Zhang; Xinlan Yang; Chaowei Zhou; Haiping Liu [0000-0002-6280-0850]; Rongzhu Zhou [0009-0003-6934-8224];

Abstract

Background

The grass carp (*Ctenopharyngodon idella*) is a large herbivorous freshwater fish belonging to the Cyprinidae family. It is widely cultivated as a food source in China and is renowned as one of the Four Great Domestic Fishes. Despite its economic importance, the published genome assemblies of grass carp remain incomplete due to gaps, thereby hindering molecular research and genetic improvement.

Results

In this study, we report the assembly of a telomere-to-telomere (T2T) gap-free genome of the grass carp with total length of 890,918,310 bp for 24 chromosomes without gaps, representing the highest completeness and assembly quality to date. Our assembly contains 27,446 protein-coding genes and 93.04% of all were annotated with multiple databases, with 48 telomeres and 24 centromeres characterized. Gap-free reference genome enable us study the structure of centromeres and identify conserved centromere-specific satellite motifs for grass carp. Furthermore, we identified 108 gene-related gaps across 12 chromosomes and 38 structural variations across 17 chromosomes in this T2T assembly.

Conclusions

The validated gap-free genome provides invaluable resource for future genomic studies grass carp, offering new insights into its genetic architecture and evolutionary dynamics.

Keywords: Grass carp; telomere-to-telomere genome; Phylogenomic

Introduction

Grass carp (*Ctenopharyngodon Idella*) (NCBI:txid7959; marinespecies.org:taxname:154314), a prominent member of the subfamily *Leuciscinae* in the *Cyprinidae* family, is distinguished by its adaptability, rapid growth, and large size [1, 2]. Its broad temperature tolerance has enabled its widespread distribution, particularly in the Yangtze, Pearl, and Heilongjiang river basins in China [3]. Recognized as one of China's "Four Domesticated Fish" in freshwater aquaculture, grass carp has a rich history of over 1700 years of cultivation within China. Since the 1980s, its aquaculture has expanded internationally to countries such as the United States, Mexico, India, and Hungary, establishing it as a valuable species in global aquaculture [4]. Grass carp also holds considerable economic importance, serving as an abundant source of high-quality protein and essential nutrients [3]. By 2023, global production of grass carp reached 5.94 million tons, making it the most extensively farmed freshwater fish both in China and worldwide.

One of the few herbivorous species in freshwater aquaculture, grass carp is uniquely adapted to a plant-based diet, a dietary trait essential to its impressive growth and adaptive success [5]. During its transition from larvae to herbivorous adults, grass carp undergo significant increases in body weight, length, and intestinal length [5]. These physiological changes correspond with genetic adaptations involving circadian rhythms, lipid synthesis, and metabolic pathways, which enhance nutrient absorption and utilization from plant-based sources. These adaptive traits underscore the importance of grass carp in aquaculture and suggest potential avenues for further research into its genetic and physiological mechanisms for growth and nutrient efficiency [1, 6].

With advances in long-read whole-genome sequencing technologies such as PacBio HiFi and Oxford Nanopore, along with the continuous development and refinement of genome assembly software, many plants and animals—such as *Homo sapiens* [7, 8], *Zea mays* [9], and *Oryza sativa* [10]—have now achieved chromosome-level T2T (telomere-to-telomere) genome assemblies. Several fish species, including *Mastacembelus armatus* [11], *Clarias gariepinus* [12], *Rhinogobio nasutus* [13], have also been sequenced to a chromosome-level T2T genome assembly. Although the grass carp genome was assembled using PacBio HiFi reads combined with Hi-C data, achieving a contig N50 of 19.3 Mb [14], a fully complete, high-quality chromosome-level genome assembly remains unavailable. Such a resource would be invaluable for advancing the study of biological functions, trait selection, and evolutionary research in this species.

In this study, we generated a telomere-to-telomere (T2T) reference genome assembly of grass carp using DNB-T7 short reads, PacBio HiFi long reads, Hi-C technology reads, and Oxford Nanopore Technologies (ONT) ultra-long reads. We compared our assembly with the latest published grass carp genome versions, highlighting the differences and improvements, and conducted a detailed analysis of centromeric regions. Comparative genomic analysis with 11 other species allowed us to identify species-specific genes. This comprehensive, chromosome-scale genome provides a robust foundation for future research into grass carp genetics, functional gene discovery, and the evolutionary genomics of teleost fishes.

Methods

Sample collection and sequencing

A wild female *C. idella* collected from Hunan Fisheries Science Institute, Changsha, Hunan, China, was used in construction of the reference genome. Genomic DNA of *C. idella* was extracted from muscle tissue using the cetyltrimethylammonium bromide (CTAB) method for sequencing library construction. Following the standard protocols of the Pacific Biosciences, DNA libraries for single-molecule real-time PacBio genome sequencing were constructed and circular consensus sequencing was performed using a PacBio Sequel IIe platform (RRID:SCR_017990) for high-fidelity (HiFi) reads. ONT ultra-long libraries were constructed and sequenced on Oxford Nanopore promethION platform (RRID:SCR_017987) for ultra-long reads. Short-read libraries of *C. idella* were constructed according to BGI DNBSEQ-T7 (RRID:SCR_017981) standard protocol, and paired-end reads (2 × 150 bp) were sequenced on an DNB-T7 platform. With default parameters, raw PacBio subreads were filtered and corrected using the pbccs pipeline.

A Hi-C library was constructed using muscle tissue of *C. idella*, which were fixed in 1% formaldehyde for crosslinking. Cells were lysed using a Dounce homogenizer and digested using the Hind III restriction enzyme. The DNA ends were filled and labeled with biotin and the filled-in Hind III sites were ligated to form Nhe I sites. Complexes with the biotin-labeled ligation products were purified and sheared, and the biotinylated Hi-C ligation products were pulled down and used to construct Hi-C library to obtain 2 × 150 bp paired-end reads using BGI DNBSEQ-T7.

Genome assembly

The HiFi, ultra-long ONT and Hi-C reads were integrated to produce the T2T assembly using Hifiasm (v. 0.19.9-r616, RRID:SCR_021069) with the parameters of --ul and verkko (v. 2.2) with default parameters, respectively [15, 16]. The HiFi reads and Hi-C reads were also assembled using Hifiasm with default parameters. The ultra-long ONT reads were assembled using nextDenovo (v. 2.5.2, RRID:SCR_025033) with parameters (read_cutoff = 1k, blocksize = 1g, nextgraph_options = -a 1) [17]. These assemblies were evaluated, and the best primary assembly generated by Hifiasm (HiFi+ONT+Hi-C) was selected and then subsequently anchored onto chromosomes using Hi-C reads. To further obtain the haplotype-resolved genome, the haplotype assemblies from verkko (HiFi+ONT+HIC) were also selected for scaffolding using Hi-C reads.

Contig sequences were clustered into 24 chromosomal groups using ALLHiC (v. 0.9.8, RRID:SCR_022750) through agglomerative hierarchical clustering [18]. Within each group, contig sequencing and orientation were performed using ALLHi-C, followed by 3D-DNA (v. 180419, RRID:SCR_017227) [19] and Juicer (v. 1.6, RRID:SCR_017226) [20] to convert interaction data into binary files. Manual adjustments were carried out using Juicebox (v. 1.11.08, RRID:SCR_021172) [21].

Telomere repair was conducted using Winnowmap (v. 1.11, RRID:SCR_025349) [22], aligning ONT reads to the reference genome and focusing on reads within 50 bp of chromosome ends. Telomeric repeat (CCCTAA/TTAGGG) were identified, and the most frequent read was designated as the reference for Medaka consensus reassembly. The consensus was aligned to chromosomes using MUMmer (v. 3.1, RRID:SCR_018171) and replaced at the chromosome ends if identity exceeded 80% [23]. Gap filling was accomplished using

Winnowmap to align gap-filling data (in the order of other assemblies > ONT reads > HiFi reads) to regions containing N's in the genome and error correction was performed on filled gap regions by Winnowmap [24] using HiFi reads (>=10 kb). Finally, we obtained the primary and two haplotype T2T assembles, and evaluated these genomes using different methods, including BUSCO, short reads mapping, Genome Continuity Inspector (GCI) score and so on. Minimap2 (v. 2-2.28, RRID:SCR_0185500) [25] and Winnowmap were used to align ONT reads and HiFi reads to genome assembly, and then GCI score was calculated by GCI [26].

Genome annotation

Repeat sequences were identified using a combination of tools to ensure comprehensive detection. RepeatModeler (v. 2.0.4, RRID:SCR_015027) [27] was utilized to predict repeat models based on the genome sequence, while LTR_FINDER (v. 1.07, RRID:SCR_015247) [28] identified long terminal repeat (LTR) sequences. The results from LTR_FINDER were processed with LTR_retriever (v. 2.9.0, RRID:SCR_017623) [29] to eliminate redundancy and construct a *de novo* repeat library. This library was merged with the RepBase database (v. 20181026, RRID:SCR_021169) and analyzed using RepeatMasker (v. 4.0.9, RRID:SCR_012954) [30] to predict repeat sequences. Additionally, RepeatProteinMask was employed to identify TE_protein-type repeats, further enhancing the accuracy and breadth of repeat annotation.

Gene structure prediction utilizes a combination of transcriptome-based, homology-based, and *de novo* approaches. Transcriptome-based prediction reconstructed transcripts using stringtie (v. 2.1.4, RRID:SCR_016323) [31], and coding regions were identified with TransDecoder (v. 5.1.0, RRID:SCR_017647). For homology-based prediction, protein sequences from related species were aligned to the genome using tblastn (v. 2.7.1, RRID:SCR_011822), and transcripts and coding regions were refined using Exonerate (v. 2.4.0, RRID:SCR_016088). *De novo* prediction was performed on repeat-masked genomes using Augustus (v. 3.3.2, RRID:SCR_008417) [32] and Genscan (v. 1.0, RRID:SCR_013362) [33]. The predictions from these methods were integrated with MAKER (v. 2.31.10, RRID:SCR_005309) [34]. To evaluate the completeness of genome annotations, BUSCO (v. 5.2.2, RRID:SCR_015008) was employed.

Protein sequences were aligned to databases such as Uniprot, NR, and the KEGG pathway database using diamond blastp (v. 2.0.11.149) [35]. Functional and pathway information was refined using KOBAS (v. 3.0, RRID:SCR_006350) with KEGG PATHWAY annotations [36, 37]. Gene Ontology (GO) terms were derived through mappings from Uniprot. To identify conserved motifs, protein domains, and structural features, HmmScan (v. 3.3.2) [38] was used with a threshold parameter of -E 0.01. Structural RNAs were predicted with specialized tools: tRNAs were identified using tRNAscan-SE (v. 1.23, RRID:SCR_008637) [39], rRNA sequences were detected using rRNA databases, and non-coding RNAs (ncRNAs) were annotated with INFERNAL (v. 1.1.2, RRID:SCR_011809) [40] based on the Rfam database. This multi-faceted approach provided a comprehensive framework for understanding genome functionality and structure.

Identifications of centromeres

TRF (Tandem Repeat Finder) (v4.09.1, RRID:SCR_022193) [41] was utilized to search tandem repeats in

de novo mode and identify locations and monomers of centromeres by BSLtool (v.1.0,). The extracted monomer sequence was used as a library with RepeatMasker to re-scan the genome. Bedtools (v. 2.30.0, RRID:SCR_006646) [42] was used to intersect the centromeric regions and these centromeric monomers were visualized and validated by StainedGlass (v. 0.6) [43].

Gene family identification, phylogenetic inference and divergence time estimation

Gene family clustering was performed using the OrthoFinder software (v. 2.3.1, RRID:SCR_017118) [44]. In addition to the genes of *C.idella* annotated in this study, protein domains were identified for genes from the following species: *Carassius auratus*, *Cyprinus carpio*, *Chanodichthys erythropterus*, *Carassius gibelio*, *Danio rerio*, *Megalobrama amblycephala*, *Ancherythroculter*, *Aristichthys nobilis*, *Mylopharyngodon piceus*, *Hypophthalmichthys molitrix*, and *Triplophysa tibetana.* Species-specific gene families, referred to as unique gene families, were identified and analyzed for functional enrichment using the clusterProfiler package to perform Gene Ontology (GO) and Kyoto Encyclopedia of Genes and Genomes (KEGG) enrichment analyses [45].

Multiple sequence alignments of protein sequences for each single-copy gene family were performed using MUSCLE (v. 3.8.31, RRID:SCR_011812) [46]. The resulting alignments were concatenated into a supergene dataset, which was used to construct a maximum likelihood (ML) phylogenetic tree using RAxML (v. 8.2.10, RRID:SCR_006086) with the model PROTGAMMAWAG [47]. Phylogenetic trees were generated for *C. idella* and 11 other species (*A. nigrocauda*, *C. auratus*, *C. carpio*, *C. erythropterus*, *C. gibelio*, *D. rerio*, *M. amblycephala*, *A. nobilis*, *M. piceus*, *H. molitrix*, and *T. tibetana*) based on shared single-copy genes. The species tree was rooted using *A. nigrocauda* and served as input for the MCMCTree program in PAML (RRID:SCR_014932) to construct an ultrametric tree [48]. Secondary calibration points were based on the divergence time between *A. nigrocauda* and *D. rerio* (41.7-68.9 million years ago), as derived from the TimeTree database.

Positive selection analysis

The single-copy orthologous genes identified between *C. carpio* and *A. nobilis* were used for positive selection analysis using WGDI (v. 0.74) [49]. Protein sequences for these genes were aligned using MUSCLE (v. 3.8.1551, RRID:SCR_011812) [50], and the Ka/Ks values were calculated using yn00 module of WGDI. A Ka/Ks ratio > 1 indicates significant positive selection. For these genes, GO and KEGG enrichment analyses were also performed using the clusterProfiler package (v. 4.0, RRID:SCR_016884) [45].

Results

A T2T gapless reference genome for *C. idella*

Various high-coverage sequencing reads were used to develop a gapless genome assembly for *C. idella*. We generated 58.86 Gb (~73×, N50 = 17.4 kb) PacBio HiFi long reads, 73.20 Gb (~91×, N50 = 100.9 kb) Oxford Nanopore Technology (ONT) ultra-long reads and 149.61 Gb (~185×) high-throughput chromatin

conformation (Hi-C) sequencing reads, along with 129.10 Gb paired-end reads (159×) (Table S1). The genome size was estimated to be 808 Mb with a heterozygosity rate of 0.49% by 19 k-mer analysis (Fig. 1A). These various types of sequencing reads were assembled and integrated by four strategy using various computational tools, including Hifiasm (HiFi, ONT ultra-long and Hi-C), verkko (HiFi, ONT ultra-long and Hi-C), Hifiasm (HiFi and Hi-C) and NextDenovo (ONT ultra-long) [51, 52]. After evaluating these different assemblies, the best assembly, which utilized Hifiasm in combination with HiFi, ONT ultra-long reads, and Hi-C data, was selected as the backbone of the T2T assembly and the remaining genome assembles were used to fill in gaps or patch telomeres (Table S2). The optimal contig assembly spanned 893 Mb with a contig N50 of 35.87 Mb, encompassing 44 telomeres (defined as >200 copies of telomeric repeat units, CCCATTT/TTTAGGG) located at one or both ends of 24 contigs. Of these, 18 contigs were classified as telomere-to-telomere (T2T), meaning they featured complete, gap-free sequences extending from one telomeric end to the other. We used the ALLHi-C to generate chromosomal interaction maps with Hi-C reads, which demonstrated all 24 chromosomes were gap free. After we added the rDNA arrays and performed telomere patching, the final T2T gapless assembly of the *C. idella* genome (CyT2T) was 890,918,310 bp on 24 gap-free chromosomes with 48 telomeres. The final chromosome ID and orientation of CyT2T were adjusted in accordance with published version (GCF_019924925.1_HZGC01).

We further performed extensive validations to ensure the accuracy and completeness of the CyT2T assembly in multiple ways. Firstly, Hi-C chromatin interaction maps showed strong consistency across all chromosomes, confirming their accurate arrangement and orientation (Fig. 1B). To estimate the base accuracy, short reads and HiFi reads were mapped to the CyT2T genome, with a mapping rate of 99.88% and 100.00%, respectively. Finally, we evaluated the genomic completeness by BUSCO at 99.1% (3607 out of 3640 in actinopterygii_odb10) and the Merqury-estimated quality value using short reads at ~49.37 (Table S3). The T2T genome achieved an overall GCI score of over 99.99%, with most chromosomes reaching 100%. Collectively, these results show that our final version of the CyT2T gap-free genome has the highest reliability and quality.

Obtainment of two haploid genomes

The haplotype-resolved assembly process successfully produced two distinct haploid genomes, designated HapA and HapB, each comprising a complete, gap-free set of chromosomes (Fig. 2 and Table S4). To evaluate their quality, we analyzed the assemblies using a combination of second-generation (next-generation sequencing, NGS) and third-generation (long-read) sequencing data. This assessment yielded mapping rates and genome coverage exceeding 99% for both haploid genomes (Table S5). High mapping rates indicate precise sequence reconstruction, while near-complete coverage confirms the absence of significant gaps or unassembled regions. Additionally, the assemblies achieved an exceptional quality value of ≥ 50, the highest among comparable haplotype-resolved genomes (Table S6). A quality value of 50 corresponds to an error rate of ≤ 0.001%, equivalent to no more than one base call error per 100,000 base pairs. This unparalleled accuracy highlights the superior quality of HapA and HapB, positioning them as a new benchmark for haplotype-resolved genome assemblies.

To further validate the assembly accuracy, we employed the genome continuity inspector (GCI) tool, a quantitative measure of assembly quality based on multiple alignment algorithms. The GCI analysis revealed exceptional assembly continuity for both HapA and HapB. For each chromosome in both haploid genomes, the observed N50 values closely matched the expected N50 values, resulting in GCI scores consistently exceeding 99.99%, and often reaching 100% (Table S7). These high GCI scores, coupled with the previously mentioned high mapping rates, genome coverage, and quality values, provide compelling evidence for the accuracy and completeness of our haplotype-resolved assemblies.

Genome annotation

Using an integrated gene annotation pipeline that combined three complementary approaches—*de novo* prediction, homologous gene prediction, and RNA-seq-based prediction—we employed four specialized tools: Genscan, AUGUSTUS, Exonerate, and TransDecoder. This comprehensive strategy identified 27,446 protein-coding genes. The BUSCO (Benchmarking Universal Single-Copy Orthologs) assessment revealed a completeness score of 97.5% for the gene set, comprising 96.2% single-copy genes and 1.3% duplicated genes (Fig. 1C). Functional annotation of these genes was highly successful, with 93.04% of them mapped to entries in six major databases: UniProt, Pfam, GO (Gene Ontology), KEGG (Kyoto Encyclopedia of Genes and Genomes), KOG (Eukaryotic Orthologous Groups), and NR (Non-Redundant Protein Database) (Tables S8-S9). The protein-coding genes exhibited an average coding sequence length of 1,630 base pairs (bp) and an average of 9.51 exons per gene (Table S8). In addition to protein-coding genes, our analysis identified 476.78 Mb of repetitive sequences, which constitute 53.52% of the genome (Table S10). Among these, class-II transposable elements (TEs), specifically DNA transposons, were the most abundant, accounting for 29.86% of the genome with a total length of 266,062,988 bp. Within class-I TEs (retrotransposons), short interspersed nuclear elements (SINEs) were the second-largest contributor, representing 4.87% of the genome and spanning 43.36 Mb (Figure S1). Furthermore, we predicted various types of non-coding RNA (ncRNA) sequences, which are critical for regulatory functions. In total, we identified 2,964 microRNAs (miRNAs), 9,322 ribosomal RNAs (rRNAs), 10,097 transfer RNAs (tRNAs), and 1,734 small nuclear RNAs (snRNAs), collectively covering approximately 2.54 Mb of the genome (Table S11).

To ensure the robustness of our assembly, we extended the annotation and prediction analyses to both haplotype genomes, HapA and HapB. We assessed repetitive sequences, gene numbers, BUSCO completeness, and ncRNA content for each haplotype. The high degree of similarity observed between HapA and HapB across these metrics underscores the consistency and accuracy of our haplotype-resolved genome assemblies (Table S12).

Correction of structural variations in the T2T genome assembly

Compared with the published version (GCF_019924925.1_HZGC01) reference genome, the major improvement in our assembly is that all 150 gaps are filled (Table S13). We performed a chromosomal synteny analysis between the assembled *C. idella* genome and *Danio rerio* genome, confirming a strong chromosomal

synteny relationship between them (Figure S2). Meanwhile, we identified and corrected 38 genomic variations, which span four types of structural variations: INV (chromosomal segments inverted relative to their normal orientation), TRANS (chromosomal segments relocated from their original positions to new locations), INVTR (regions that have undergone both inversion and translocation), and INVDP (duplicated chromosomal segments inverted relative to their normal orientation) (Table S14). These variations were distributed across 17 chromosomes, covering regions ranging from 1.02 Mb to 13.61 Mb (Fig. 3A). To ensure the accuracy of these corrected regions, we further examined the read coverage within these variant regions (Figure S3). Additionally, the completed T2T version of the genome also improved the existing publicly available *C. idella* genome by filling 150 gap regions. This includes the completion of gaps, which involve 108 genes across 12 chromosomes (Table S15). These results demonstrate the significant improvements made in the T2T version of the *C. idella* genome compared to the currently available version.

Analysis of centromeric monomers

The centromeres in the genome were identified and characterized based on their genomic positions, lengths, and monomer compositions. The 24 chromosomes exhibited centromeres with lengths ranging from 189,322 bp (chr17) to 501,158 bp (chr16), with an average length of approximately 340,000 bp (Fig. 3B and Table S16). Detailed annotations revealed distinct patterns of centromeric organization across chromosomes. For instance, chr2 had a centromere spanning 448,577 bp (24,734,939-25,183,515), while chr16 contained the largest centromere, spanning 501,158 bp (8,483,580-8,984,737). Conversely, chr17 had the smallest centromere at 189,322 bp (10,882,091-11,071,412). The findings provide a comprehensive understanding of the centromeric architecture, revealing variation in centromere length and organization among chromosomes, highlighting their unique structural characteristics.

Expansion and contraction of gene families and phylogenetic inference

We investigated the expansion and contraction of gene families during the evolution of Cyprinidae. Our focus was on the impact of polyploidization events in Cyprinidae, which corresponded to 9,740 expanded and 473 contracted gene families in the *Cyprininae* subfamily (including *Cyprinus carpio*, *Carassius auratus*, and *Carassius gibelio*) (Fig. 4A, Tables S17-S18). Additionally, we examined four major Asian domestic carps: grass carp (*C. idella*), black carp (*Mylopharyngodon piceus*), bighead carp (*Aristichthys nobilis*), and silver carp (*Hypophthalmichthys molitrix*). We identified 84 uniquely expanded and 1,612 contracted gene families in the *Hypophthalmichthyinae* subfamily for *A. nobilis* and *H. molitrix*, and 23 uniquely expanded and 541 contracted gene families in the *Leuciscidae* subfamily for *M. piceus* and *C. idella*. Notably, grass carp, a herbivorous fish, exhibited 317 uniquely expanded and 763 contracted gene families. Additionally, we classified the gene family clusters into four categories: single-copy orthologs, multiple-copy orthologs, species-specific genes (unique paralogs), and other orthologs. In *C. idella*, we identified 18,686 gene families, including 10,112 single-copy orthologs, 2,639 multiple-copy orthologs, and 2,493 species-specific genes (Fig. 4B).

Using single-copy orthologous genes, we constructed a phylogenetic tree, which revealed that *Danio rerio*

diverged from other Cyprinidae species approximately 79.1 million years ago (MYA). Furthermore, based on the phylogenetic tree and fossil calibration, we estimated that the divergence between *C. idella* and *M. piceus* occurred around 10.7 MYA, while the divergence between *A. nobilis* and *H. molitrix*, both members of the "Four Major Chinese Carps," occurred approximately 6.7 MYA (Fig. 4A).

GO and KEGG enrichment analyses revealed that expanded gene families in grass carp are associated with sensory and food-related activities, including olfactory receptor activity (GO: 0004984), G-protein coupled receptor activity (GO: 0004930), and the G-protein coupled receptor signaling pathway (GO: 0007186) (Fig. 4A, Table S19, and Figure S4). In contrast, contracted gene families are enriched in digestion-related functions, such as serine-type endopeptidase activity (GO: 0004252) and metalloendopeptidase activity (GO: 0004222) (Fig. 4A, Table S20, and Figure S4). These findings suggest a potential association between the expansion and contraction of gene families and the dietary evolution of grass carp.

Ka/Ks analysis

We calculated the Ka/Ks values between *C. carpio* and *A. nobilis* and identified 263 genes as positively selected genes (PSGs) (Ka/Ks > 1) (Table S21). GO analysis of these PSGs revealed that three genes were associated with the adaptive immune response (GO: 0002250, p-value < 0.001), four genes are linked to chemokine activity (GO: 0008009, p-value = 0.002), and two genes are related to G-protein beta-subunit binding (GO: 0031681, p-value = 0.005) (Table S22).

Reuse potential

The T2T genome of grass carp (*C. idella*), a large herbivorous freshwater fish from the Cyprinidae family, represents a groundbreaking and highly versatile genomic resource with immense potential for advancing research and practical applications. As the most economically significant freshwater aquaculture species in China and globally, grass carp is a cornerstone of food production and is revered as one of the Four Great Domestic Fishes. However, prior genome assemblies of this species have been hampered by gaps and incompleteness, limiting progress in molecular research and genetic enhancement. This telomere-to-telomere (T2T) gap-free assembly, spanning 890,918,310 bp across 24 chromosomes, achieves unprecedented completeness and quality, providing an exceptional foundation for exploring the species' genomic landscape and supporting its genetic improvement.

This T2T assembly encompasses 27,446 protein-coding genes, with 93.04% annotated using multiple databases, alongside the characterization of 48 telomeres and 24 centromeres. The gap-free reference genome enables detailed investigation of centromere structures, revealing conserved centromere-specific satellite motifs unique to grass carp. This resource empowers researchers to probe evolutionary dynamics and functional variations, potentially identifying genes linked to traits like growth rate or disease resistance—crucial for aquaculture breeding programs. By serving as a comprehensive reference, this T2T genome paves the way for future studies, such as haplotype-resolved analyses or the development of a Cyprinidae pan-genome, to further illuminate adaptive traits and species divergence.

Discussion

Grass carp, as the most economically important fish species in both China and worldwide, holds significant potential for advancements in genomic breeding technologies, such as whole-genome selection and other molecular breeding approaches [1, 2]. In this study, we present the first T2T genome assembly of grass carp. To achieve this, we employed an integrated sequencing approach, utilizing high-depth DNB-T7 short reads, PacBio HiFi long reads, and Hi-C technology reads, alongside ONT ultra-long reads. This diverse approach allows for the generation of a high-quality, chromosome-scale reference genome, resolving a number of previously reported assembly errors.

Compared to the previously published chromosome-level genome [14], our assembly corrects 38 assembly errors, fills 150 genomic gaps, and completes the assembly of previously unresolved telomeric and centromeric regions. This refinement of the grass carp genome offers a more comprehensive and accurate representation of its genomic architecture. The improved genome assembly provides a robust resource for more accurate surveys of germplasm resources across different regions, population genetic analyses, and breeding studies in grass carp, potentially accelerating the development of genetically improved strains for aquaculture.

Beyond its utility in breeding and genomic research, our study also contributes to a deeper understanding of the evolutionary mechanisms shaping the Cyprinidae family, particularly in terms of gene family expansions and contractions. These analyses revealed distinct patterns in gene family dynamics, which are likely linked to the unique ecological niche and dietary preferences of grass carp. For example, we identified specific gene family expansions related to sensory perception and food-related activities, such as olfactory receptors and G-protein coupled receptors. These findings suggest that grass carp, as a herbivorous species, may have evolved specialized molecular mechanisms to adapt to its unique feeding behavior.

This comprehensive genome also provides a solid foundation for further comparative genomics studies in the Cyprinidae family and offers new opportunities for exploring the evolutionary relationships between grass carp and other species. With more detailed genomic data, we can now better understand how grass carp has evolved to thrive in diverse aquatic environments and how its genome may have adapted to the specific demands of herbivorous feeding.

Overall, this study not only enriches our understanding of the grass carp's biology and evolution but also contributes to the broader field of teleost fish genomics. By leveraging the full potential of this high-quality genome, we can further explore the genetic underpinnings of key traits in grass carp, facilitating improvements in breeding programs and providing valuable insights into the broader evolutionary dynamics of freshwater fish.

Supplementary

Data Availability

The genomic sequence and RNA-seq data of *C. idella* generated by this study were deposited into in NCBI under the accession number PRJNA1251791 and NGDC (National Genomics Data Center) database under the accession number PRJCA036158. The assembled genome sequences and annotation information have been

submitted in NGDC under accession number PRJCA036158. All additional supporting data are available in the *GigaScience* repository, GigaDB [53].

Abbreviations

T2T: telomere-to-telomere; ONT: Oxford Nanopore Technologies; BUSCO: Benchmarking Universal Single-Copy Orthologs; HiFi: high fidelity; TEs: transposable elements; SINEs: short interspersed nuclear elements; miRNAs: microRNAs; rRNAs: ribosomal RNAs; tRNAs: transfer RNAs; snRNAs: small nuclear RNAs; INV: chromosomal segments inverted relative to their normal orientation; TRANS: chromosomal segments relocated from their original positions to new locations; INVTR: regions that have undergone both inversion and translocation; INVDP: duplicated chromosomal segments inverted relative to their normal orientation; MYA: million years ago; LTR: identified long terminal repeat; GO: Gene Ontology; ncRNAs: non-coding RNAs; TRF: Tandem Repeat Finder; KEGG: Kyoto Encyclopedia of Genes and Genomes; ML: maximum likelihood; PSGs: positively selected genes.

ACKNOWLEDGEMENTS

We thank for the funding provided by Guangzhou Key R&D Program (2024B03J1082), Tibet Autonomous Region financial project (survey, prevention and control of non-native fish), National Natural Science Foundation of China (NSFC) Joint Fund Priority Support Program (U23A20249) for this study.

AUTHOR CONTRIBUTIONS

Fei Liu: Data curation; formal analysis; investigation; methodology; software; writing-original draft.Yuan Li：Data analysis； methodology. Guishuang Wang and Dong Zhang: Data curation; methodology; project administration; resources. Xinlan Yang: Project administration; writing-review and editing. Chaowei Zhou: Data curation; software. Haiping Liu: Conceptualization; funding acquisition; methodology; project administration; resources; supervision.

Disclosure and competing interests statement

The authors have declared that no competing interests exist.

References

1. Ding, L. (2017). Grass carp, Ctenopharyngodon idella. In Handbook of Nutrient Requirements of Finfish (1991). (CRC Press), pp. 89-96.
2. Wang, Y., Liu, W., Li, Z., Qiu, B., Li, J., Geng, G., Hu, B., Liao, A., Cai, Y., Wen, M., et al. (2024). Improvement and application of genetic resources of grass carp (Ctenopharyngodon idella). Reproduction and Breeding *4*, 126-133.
3. Zhao, Y., Zhang, L., Wang, C., and Xie, C. (2020). Biology and ecology of grass carp in China: a review

and synthesis. North American Journal of Fisheries Management *40*, 1379-1399.

4. Li, L., Balto, G., Xu, X., Shen, Y., and Li, J. (2023). The feeding ecology of grass carp: a review. Reviews in Aquaculture *15*, 1335-1354.

5. Zhao, H., Xia, J., Zhang, X., He, X., Li, L., Tang, R., Chi, W., and Li, D. (2018). Diet affects muscle quality and growth traits of grass carp (Ctenopharyngodon idellus): a comparison between grass and artificial feed. Frontiers in physiology *9*, 283.

6. Hu, F., Zhong, H., Wu, C., Wang, S., Guo, Z., Tao, M., Zhang, C., Gong, D., Gao, X., Tang, C., et al. (2021). Development of fisheries in China. Reproduction and Breeding *1*, 64-79.

7. Miga, K.H., Koren, S., Rhie, A., Vollger, M.R., Gershman, A., Bzikadze, A., Brooks, S., Howe, E., Porubsky, D., and Logsdon, G.A. (2020). Telomere-to-telomere assembly of a complete human X chromosome. Nature *585*, 79-84.

8. Nurk, S., Koren, S., Rhie, A., Rautiainen, M., Bzikadze, A.V., Mikheenko, A., Vollger, M.R., Altemose, N., Uralsky, L., and Gershman, A. (2022). The complete sequence of a human genome. Science *376*, 44-53.

9. Chen, J., Wang, Z., Tan, K., Huang, W., Shi, J., Li, T., Hu, J., Wang, K., Wang, C., and Xin, B. (2023). A complete telomere-to-telomere assembly of the maize genome. Nature genetics *55*, 1221-1231.

10. Huang, X. (2023). A complete telomere-to-telomere assembly provides new reference genome for rice. Molecular Plant *16*, 1370-1372.

11. Xue, L., Gao, Y., Wu, M., Tian, T., Fan, H., Huang, Y., Huang, Z., Li, D., and Xu, L. (2021). Telomere-to-telomere assembly of a fish Y chromosome reveals the origin of a young sex chromosome pair. Genome biology *22*, 203.

12. Nguinkal, J.A., Zoclanclounon, Y.A., Brunner, R.M., Chen, Y., and Goldammer, T. (2024). Haplotype-resolved and near-T2T genome assembly of the African catfish (Clarias gariepinus). Scientific Data *11*, 1095.

13. Jiang, C., Du, Y., Lou, Z., Zhang, Y., and Wang, T. (2025). Telomere-to-telomere reference genome of Rhinogobio nasutus, an endangered endemic fish from the Yellow River. Scientific Data *12*, 462.

14. Wu, C.-S., Ma, Z.-Y., Zheng, G.-D., Zou, S.-M., Zhang, X.-J., and Zhang, Y.-A. (2022). Chromosome-level genome assembly of grass carp (Ctenopharyngodon idella) provides insights into its genome evolution. BMC genomics *23*, 271.

15. Chen, Y., Nie, F., Xie, S.-Q., Zheng, Y.-F., Dai, Q., Bray, T., Wang, Y.-X., Xing, J.-F., Huang, Z.-J., Wang, D.-P., et al. (2021). Efficient assembly of nanopore reads via highly accurate and intact error correction. Nature communications *12*, 60.

16. Rautiainen, M., Nurk, S., Walenz, B.P., Logsdon, G.A., Porubsky, D., Rhie, A., Eichler, E.E., Phillippy, A.M., and Koren, S. (2023). Telomere-to-telomere assembly of diploid chromosomes with Verkko. Nature biotechnology *41*, 1474-1482.

17. Hu, J., Wang, Z., Sun, Z., Hu, B., Ayoola, A.O., Liang, F., Li, J., Sandoval, J.R., Cooper, D.N., and Ye, K. (2024). NextDenovo: an efficient error correction and accurate assembly tool for noisy long reads.

Genome biology *25*, 107.

18. Zhang, X., Zhang, S., Zhao, Q., Ming, R., and Tang, H. (2019). Assembly of allele-aware, chromosomal-scale autopolyploid genomes based on Hi-C data. Nature plants *5*, 833-845.
19. Dudchenko, O., Batra, S.S., Omer, A.D., Nyquist, S.K., Hoeger, M., Durand, N.C., Shamim, M.S., Machol, I., Lander, E.S., and Aiden, A.P. (2017). De novo assembly of the Aedes aegypti genome using Hi-C yields chromosome-length scaffolds. Science *356*, 92-95.
20. Durand, N.C., Shamim, M.S., Machol, I., Rao, S.S., Huntley, M.H., Lander, E.S., and Aiden, E.L. (2016). Juicer provides a one-click system for analyzing loop-resolution Hi-C experiments. Cell systems *3*, 95-98.
21. Durand, N.C., Robinson, J.T., Shamim, M.S., Machol, I., Mesirov, J.P., Lander, E.S., and Aiden, E.L. (2016). Juicebox provides a visualization system for Hi-C contact maps with unlimited zoom. Cell systems *3*, 99-101.
22. Jain, C., Rhie, A., Zhang, H., Chu, C., Walenz, B.P., Koren, S., and Phillippy, A.M. (2020). Weighted minimizer sampling improves long read mapping. Bioinformatics *36*, i111-i118.
23. Marçais, G., Delcher, A.L., Phillippy, A.M., Coston, R., Salzberg, S.L., and Zimin, A. (2018). MUMmer4: A fast and versatile genome alignment system. PLoS computational biology *14*, e1005944.
24. Jain, C., Rhie, A., Hansen, N.F., Koren, S., and Phillippy, A.M. (2022). Long-read mapping to repetitive reference sequences using Winnowmap2. Nature methods *19*, 705-710.
25. Li, H. (2021). New strategies to improve minimap2 alignment accuracy. Bioinformatics *37*, 4572-4574.
26. Chen, Q., Yang, C., Zhang, G., and Wu, D. (2024). GCI: a continuity inspector for complete genome assembly. Bioinformatics *40*, btae633.
27. Flynn, J.M., Hubley, R., Goubert, C., Rosen, J., Clark, A.G., Feschotte, C., and Smit, A.F. (2020). RepeatModeler2 for automated genomic discovery of transposable element families. Proceedings of the National Academy of Sciences of the United States of America *117*, 9451-9457.
28. Xu, Z., and Wang, H. (2007). LTR_FINDER: an efficient tool for the prediction of full-length LTR retrotransposons. Nucleic acids research *35*, W265-268.
29. Ou, S., and Jiang, N. (2018). LTR_retriever: A Highly Accurate and Sensitive Program for Identification of Long Terminal Repeat Retrotransposons. Plant physiology *176*, 1410-1422.
30. Tempel, S. (2012). Using and understanding RepeatMasker. Methods in molecular biology *859*, 29-51.
31. Shumate, A., Wong, B., Pertea, G., and Pertea, M. (2022). Improved transcriptome assembly using a hybrid of long and short reads with StringTie. PLoS Comput Biol *18*, e1009730.
32. Stanke, M., Diekhans, M., Baertsch, R., and Haussler, D. (2008). Using native and syntenically mapped cDNA alignments to improve de novo gene finding. Bioinformatics *24*, 637-644.
33. Burge, C., and Karlin, S. (1997). Prediction of complete gene structures in human genomic DNA. Journal of molecular biology *268*, 78-94.
34. Holt, C., and Yandell, M. (2011). MAKER2: an annotation pipeline and genome-database management tool for second-generation genome projects. BMC bioinformatics *12*, 491.

35. Buchfink, B., Xie, C., and Huson, D.H. (2015). Fast and sensitive protein alignment using DIAMOND. Nature methods *12*, 59-60.
36. Xie, C., Mao, X., Huang, J., Ding, Y., Wu, J., Dong, S., Kong, L., Gao, G., Li, C.Y., and Wei, L. (2011). KOBAS 2.0: a web server for annotation and identification of enriched pathways and diseases. Nucleic acids research *39*, W316-322.
37. Bu, D., Luo, H., Huo, P., Wang, Z., Zhang, S., He, Z., Wu, Y., Zhao, L., Liu, J., and Guo, J. (2021). KOBAS-i: intelligent prioritization and exploratory visualization of biological functions for gene enrichment analysis. Nucleic acids research *49*, W317-W325.
38. Eddy, S.R. (2011). Accelerated Profile HMM Searches. PLoS Comput Biol *7*, e1002195.
39. Chan, P.P., Lin, B.Y., Mak, A.J., and Lowe, T.M. (2021). tRNAscan-SE 2.0: improved detection and functional classification of transfer RNA genes. Nucleic acids research *49*, 9077-9096.
40. Nawrocki, E.P., and Eddy, S.R. (2013). Infernal 1.1: 100-fold faster RNA homology searches. Bioinformatics *29*, 2933-2935.
41. Benson, G. (1999). Tandem repeats finder: a program to analyze DNA sequences. Nucleic acids research *27*, 573-580.
42. Quinlan, A.R., and Hall, I.M. (2010). BEDTools: a flexible suite of utilities for comparing genomic features. Bioinformatics *26*, 841-842.
43. Vollger, M.R., Kerpedjiev, P., Phillippy, A.M., and Eichler, E.E. (2022). StainedGlass: interactive visualization of massive tandem repeat structures with identity heatmaps. Bioinformatics *38*, 2049-2051.
44. Emms, D.M., and Kelly, S. (2019). OrthoFinder: phylogenetic orthology inference for comparative genomics. Genome biology *20*, 238.
45. Wu, T., Hu, E., Xu, S., Chen, M., Guo, P., Dai, Z., Feng, T., Zhou, L., Tang, W., Zhan, L., et al. (2021). clusterProfiler 4.0: A universal enrichment tool for interpreting omics data. Innovation *2*, 100141.
46. Edgar, R.C. (2004). MUSCLE: multiple sequence alignment with high accuracy and high throughput. Nucleic acids research *32*, 1792-1797.
47. Stamatakis, A. (2014). RAxML version 8: a tool for phylogenetic analysis and post-analysis of large phylogenies. Bioinformatics *30*, 1312-1313.
48. Yang, Z. (2007). PAML 4: phylogenetic analysis by maximum likelihood. Molecular biology and evolution *24*, 1586-1591.
49. Sun, P., Jiao, B., Yang, Y., Shan, L., Li, T., Li, X., Xi, Z., Wang, X., and Liu, J. (2022). WGDI: A user-friendly toolkit for evolutionary analyses of whole-genome duplications and ancestral karyotypes. Mol Plant *15*, 1841-1851.
50. Edgar, R.C. (2004). MUSCLE: multiple sequence alignment with high accuracy and high throughput. Nucleic acids research *32*, 1792-1797.
51. Cheng, H., Concepcion, G.T., Feng, X., Zhang, H., and Li, H. (2021). Haplotype-resolved de novo assembly using phased assembly graphs with hifiasm. Nature methods *18*, 170-175.
52. Jiang, H., Zhuo, W., Zongyi, S., Benxia, H., Adeola Oluwakemi, A., Fan, L., Jingjing, L., José, R.S.,

David, N.C., Kai, Y., et al. NextDenovo: an efficient error correction and accurate assembly tool for noisy long reads. Genome Biology. 2024. https://doi.org/10.1186/s13059-024-03252-4

53. Liu F, Li Y, Wang G, Zhang D, Yang X, Zhou C, et al. Supporting data for "The telomere-to-telomere gapless genome of grass carp provides insights for genetic improvement" GigaScience Database. 2025. https://doi.org/10.5524/102701

Figure legend

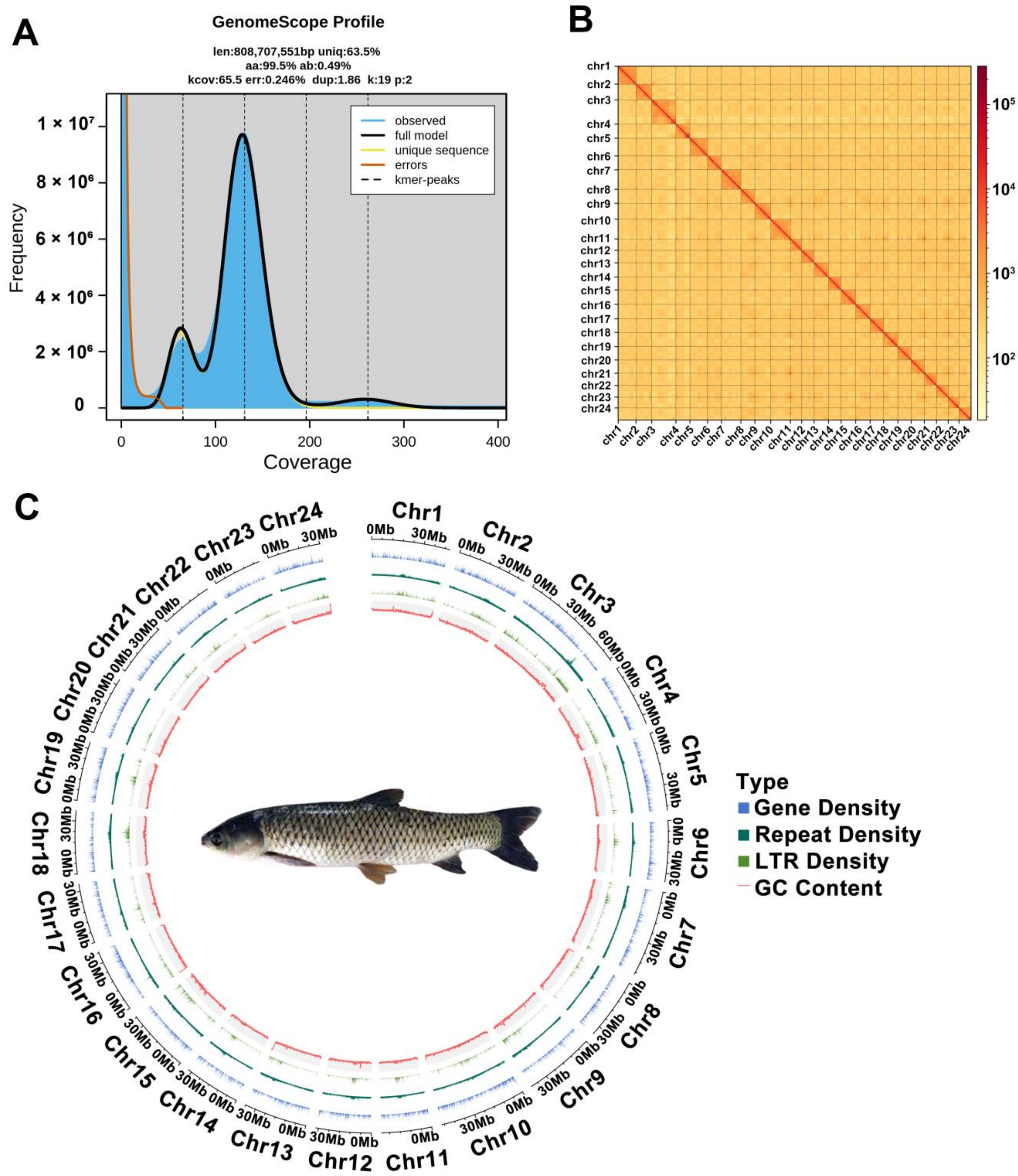

Figure 1. Genome description of *C. idella*.

(A) GenomeScope estimation of genome size and heterogeneity using a k-mer of 19.

(B) Hi-C interaction map.

(C) A Circos plot of the assembled pseudochromosomes. Densities were calculated in 100-kb windows.

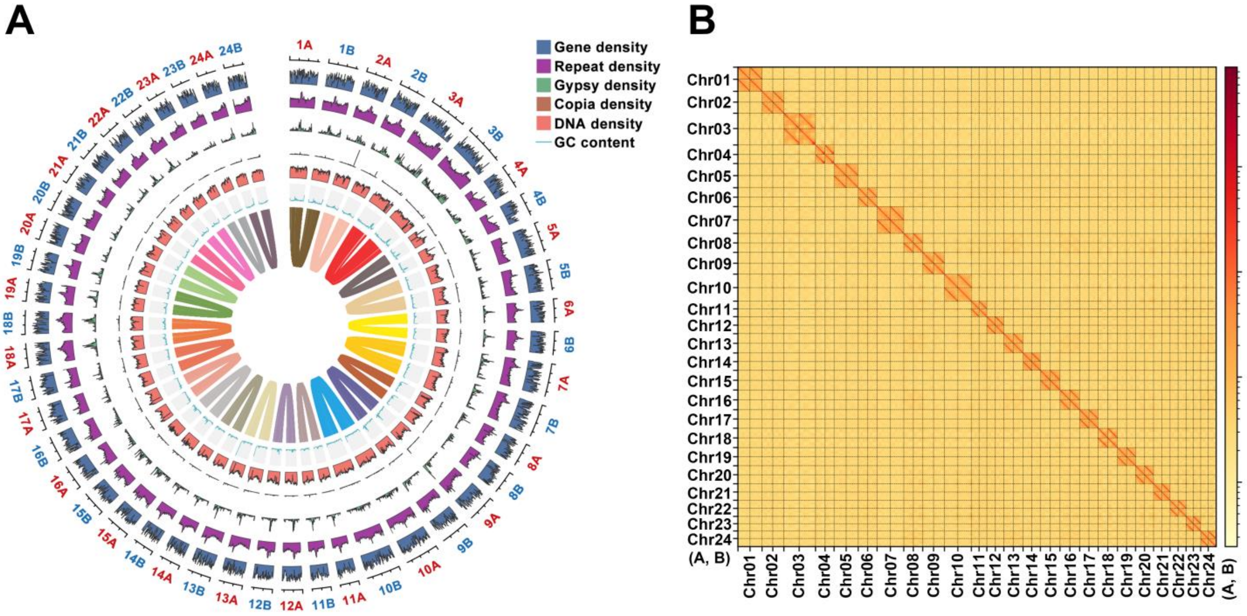

Figure 2. Haploid genomes A and B of *C. idella*.

(A) Circos plot of haploid genomes A and B. (I–VII) From outermost to innermost, concentric circles show chromosomes (I), GC content (II), gene density (III), LTR/Gypsy density (IV), LTR/Copia density (V), DNA transposon density (VI), and syntenic regions >100 kb between the A and B haplotype genomes (VII).

(B) Hi-C interaction map of haploid genomes A and B.

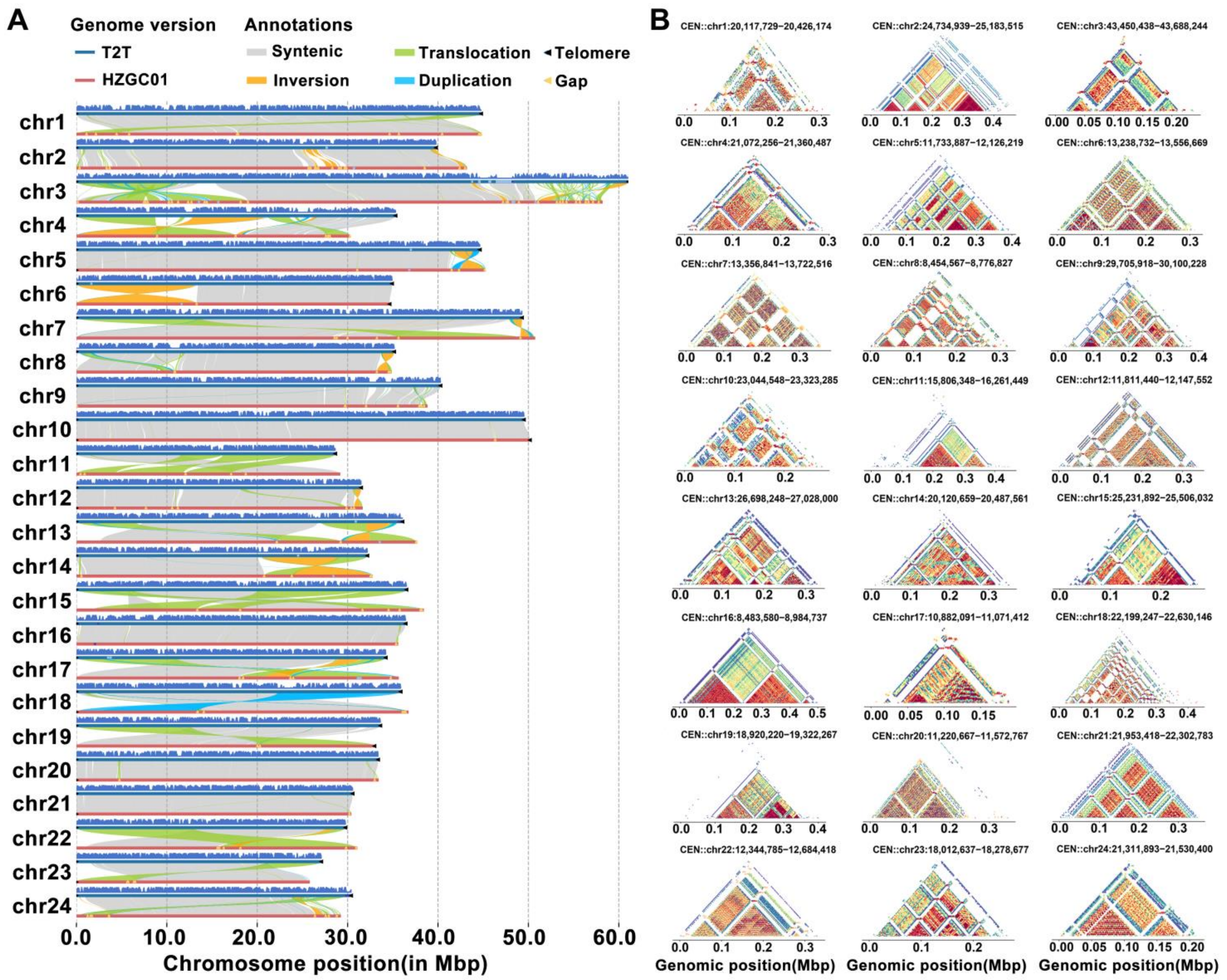

Figure 3. Comparison of the two *C. idella* genome versions.

(A) Synteny analysis of the two *C. idella* genome versions. "T2T" refers to the assembled genome obtained in this study, while "HZGC01" refers to the publicly available genome (NCBI: GCF_019924925.1).

(B) StainedGlass sequence identity heatmaps of centromeres for the T2T version.

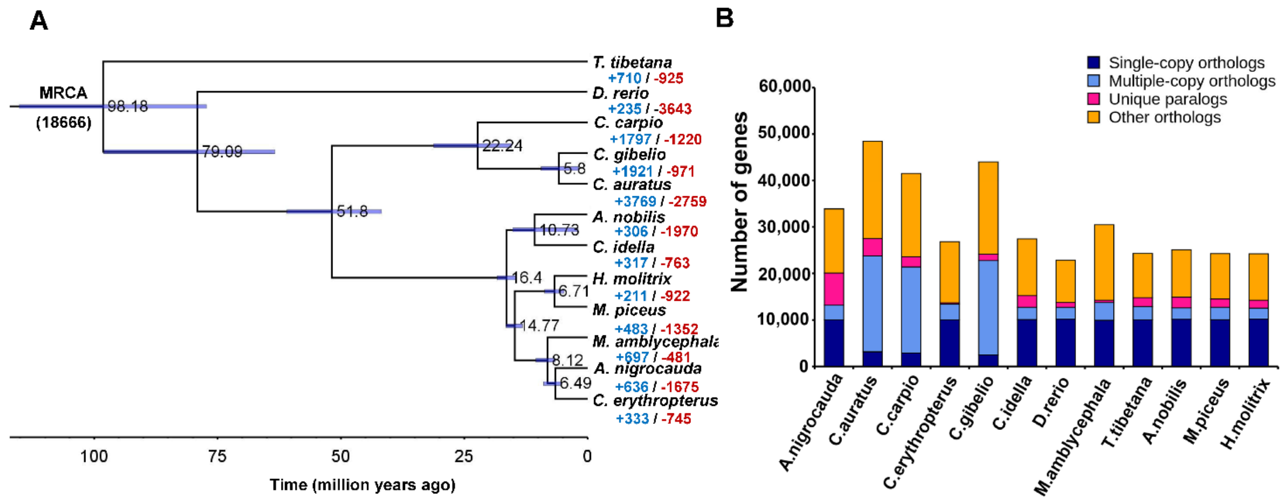

Figure 4. Comparative genomic analysis of *C. idella* and its related species.

(A) Phylogenetic tree representing the number of gene families that have expanded or contracted among 12 species. The number at the root (18,666) denotes the total number of gene families predicted in the most recent common ancestor (MRCA). The estimated divergence time (in millions of years) is shown beside the branch nodes in black. The scale on the x-axis shows the estimated divergence time for nodes. "+" indicates that gene families expanded, and "–" indicates that gene families contracted.

(B) The prediction of single-copy and multi-copy gene families across 12 species.

Table

Click here to access/download

Supplementary Material

Supplementary Tables--REV.xlsx
