# Supplementary material for: The telomere-to-telomere gapless genome of grass carp provides insights for genetic improvement
Source: Gigascience. 2025 Jun 18;14:giaf059. doi: 10.1093/gigascience/giaf059 (PMC12204074; doi:10.1093/gigascience/giaf059)
Supplement: giaf059_Authors_Response_To_Reviewer_Comments_original_submission [file giaf059_authors_response_to_reviewer_comments_original_submission.pdf]

# Author's Response To Reviewer Comments

Dear Dr. Hongfang Zhang,
Please find the revised manuscript entitled “The telomere-to-telomere gapless genome of grass carp provide insights for genetic improvement” (GIGA-D-25-00078), which we would like to submit for publication as original research in GigaScience.
The comments provided by the editors and reviewers have enabled us to further improve the quality of our manuscript. The grammar and formatting errors have been comprehensively corrected throughout the text. We have highlighted the revised sections in yellow. Detailed explanations and our point-by-point responses to each of the comments are provided in the following pages.

We are looking forward to hearing from you at your earliest convenience.

Sincerely!

Prof. Rongzhu Zhou
National Animal Husbandry Services, Beijing 100125, China

Responses to the comments of the editor:
1. Looking at this the referencing is totally messed up - as we use chronological and numerical, while it's alphabetical in your manuscript. Please redo the referencing so the copy editor doesn't mess this up. Please ensure that the reference style is followed precisely and consistently. You can refer to the author guidelines https://academic.oup.com/gigascience/pages/data_note for more details.
Answer
Thank you for your kind comments. As per your suggestion, we have revised the reference formatting accordingly.

2. In the methods section it would be useful if you add RRID details to some of the software tools and sequencers. These needed to be listed after resources in brackets. If you are citing papers for these resources, the RRID do not replace these, and both should be included. This can be included in the methods section of the paper similar to the RRIDs included here:
DNBSEQ-T7 (RRID:SCR_017981); PacBio Sequel II System (RRID:SCR_017990); PLINK (RRID:SCR_001757)
Answer
Thank you for your kind comments. We have added the RRID details for the software tools and sequencing platforms used in the Methods section of the revised manuscript.

Responses to the comments of the reviewer 1:
This manuscript presents the generation and initial characterization of a telomere-to-telomere (T2T), gapless genome assembly for the economically important grass carp, Ctenopharyngodon idella. The authors utilized a state-of-the-art combination of PacBio HiFi, Oxford Nanopore ultra-long reads, Hi-C, and Illumina sequencing, achieving impressive assembly quality metrics (T2T chromosomes, high BUSCO, QV, and GCI scores). The methods are detailed, validation is thorough, and the data availability statement is clear. While the manuscript is generally strong, a few minor points could further improve its clarity and impact.
Answer
We sincerely appreciate your valuable feedback, which has significantly enhanced the quality and clarity of our work. In the following sections, we have addressed each of your comments with detailed revisions and responses.

1.The Introduction states that Mastacembelus armatus is the only fish (at the time of writing) with a chromosome-level T2T assembly. While likely true when drafted, the T2T field moves rapidly. Please double-check if any other fish T2T assemblies (chromosome-level) have been published very recently and update the statement if necessary for accuracy at the time of publication.
Answer

Yes, we have collected information on fish species that have recently published T2T genome versions, and indeed, we found that several fish species have been sequenced. We have updated the Introduction section accordingly. The revision is as follows:
"Several fish species, including Mastacembelus armatus (Xue et al. 2021), Clarias gariepinus (Nguinkal et al. 2024), Rhinogobio nasutus (Jiang et al. 2025), have also been sequenced to a chromosome-level T2T genome assembly." (Page 3, lines 23-25)

2.The authors state they used different assembly strategies/tools (Hifiasm, verkko, NextDenovo) and selected the "best assembly" (Hifiasm with HiFi+ONT+Hi-C) as the backbone. The criteria for "best" are not explicitly stated or quantified comparatively.
Answer
Thank you for the kind comments. We have included a new Table S2 in the latest revised version to provide detailed information on the genome assemblies generated using multiple assembly strategies.
Table S2. Evaluation of contig-level genome assemblies generated by different assembly strategies.
Versions of contig-level genome assemblies Length (bp) N50 (bp) Quality Value Number BUSCO Depth (X)
Hifiasm (HIFI+HIC) 1773254753 33561093 51.38 139 C:99.1%[S:0.1%,D:99.0%] 33.19
Hifiasm (HIFI+ONT+HIC) 1772438864 35677735 51.23 95 C:99.1%[S:0.1%,D:99.0%] 33.21
Verkko (HIFI+HIC) 1780739888 18626258 50.67 669 C:99.1%[S:0.3%,D:98.8%] 33.05
Verkko (HIFI+ONT+HIC) 1777471075 35567384 50.83 243 C:99.1%[S:0.1%,D:99.0%] 33.11
*"C" represents complete BUSCOs, "S" indicates complete and single-copy BUSCOs, and "D" indicates complete but duplicated BUSCOs.

3.Title, 'genome provide insights' should be 'genome provides insights'
Answer
Thank you for the kind comments. We have revised it.

4.Page 4: The latin name should be italized. This should be checked across the whole manuscript.
Answer
We have thoroughly checked and revised the entire manuscript. Thank you for your valuable comments.

5. Page 7: The line spacing is not unified.
Answer
We have thoroughly checked and revised the entire manuscript. Thank you for your valuable comments.

Responses to the comments of the reviewer 2:
1.Grass carp (Ctenopharyngodon idella) is the most productive freshwater aquaculture species in China. Each year, a large number of studies are published on its genetic improvement, immunity, and nutrition. The newly reported T2T genome in this study provides a valuable reference for researchers working in these areas, both domestically and internationally. As a data note, this manuscript presents a relatively comprehensive analysis of the newly assembled genome. Compared to previously published grass carp genomes, this version shows notable improvements in quality, particularly in telomeric, centromeric, and other repetitive regions. I recommend acceptance after minor revision.
Answer
Thank you for the kind comments! In the latter part of this document, we have provided detailed point-by-point responses to the comments raised.

1.Sample Information: Clarify the sex (male or female) of the grass carp individual used for genome sequencing.
Answer
We selected a male grass carp as the specimen for genome sequencing. The following statement has been added to the Methods section:
"A wild female C. idella collected from Hunan Fisheries Science Institute, Changsha, Hunan, China, was used in construction of the reference genome." (page 4, lines 2-3)

2.Genome Size Discrepancy: The manuscript reports an estimated genome size of 808 Mb but a final T2T assembly size of ~891 Mb. Explain the difference (e.g., k-mer estimation vs. assembly output).
Answer

These two values represent different aspects of the genome and should not be confused. The "808 Mb" refers to the initial genome size estimated using k-mer analysis, while the "891 Mb" corresponds to the final size of the completed T2T assembly. This discrepancy highlights that genome size estimates based on k-mer analysis may not fully reflect the actual size of the genome.

3.T2T Chromosome Definition: The statement "44 telomeres... 18 of which were T2T" is unclear, given grass carp has 24 chromosomes. Elaborate on the criteria for designating chromosomes as T2T, as only 18 are labeled as such despite the T2T (gap-free) implication.
Answer
To improve clarity, we have revised the sentence as follows:
"The optimal contig assembly spanned 893 Mb with a contig N50 of 35.87 Mb, encompassing 44 telomeres (defined as >200 copies of telomeric repeat units, CCCATTT/TTTAGGG) located at one or both ends of 24 contigs. Of these, 18 contigs were classified as telomere-to-telomere (T2T), meaning they featured complete, gap-free sequences extending from one telomeric end to the other." (page 7, lines 12-16)

4.Terminology Consistency: In the Results section, "CT2T" appears—should this be "CyT2T" as used earlier? Ensure consistent terminology throughout.
Answer
Thank you for the valuable comments. We have implemented the changes.

5.Italicize the Latin name Ctenopharyngodon idella in the Methods section under "Sample collection and sequencing" (e.g., "Short-read libraries of C. idella...").
Standardize sequencing depth notation in the Results section (e.g., use "~73×," "~91×," "~185×" consistently instead of mixing "73×" and "91×").
Fix general formatting errors: remove double spaces, standardize "HiFi" (not "HIFI"), and adjust the section title "Positive Selection Analysis" to "Positive selection analysis" (capitalize only the first word).
Add line numbers to the manuscript for easier review.
Answer
Thank you for the valuable comments. We have revised the errors as described.

6.Grammatical and Clarity Improvements:
Revise "Different and high-coverage sequencing reads were used" to "Various high-coverage sequencing reads were used" for smoother phrasing.
Correct "The rest genome assembles were used" to "The remaining genome assemblies were used."
Add a subject to "After addition of the rDNA arrays and telomere patching..." (e.g., "After we added the rDNA arrays and performed telomere patching...").
Simplify "Hi-C chromatin interaction maps exhibited consistent consistency" (redundant) to "Hi-C chromatin interaction maps showed strong consistency" or similar.
Change "three genes are associated" to "were associated" for consistent past tense.
Answer
Thank you for the valuable comments. We have revised the errors as described.

Precision and Clarity:
The "GCI score of 99.9999%" seems overly precise. Round to 99.99% unless the method justifies this level of detail.
Answer
Thank you for the valuable comments. We have revised it as below:
"The T2T genome achieved an overall GCI score of over 99.99%, with most chromosomes reaching 100%."(page 7, lines 12-16)

Reference:

Jiang C, Du Y, Lou Z, Zhang Y, Wang T. 2025. Telomere-to-telomere reference genome of Rhinogobio nasutus, an endangered endemic fish from the Yellow River. Scientific Data 12(1): 462.
Nguinkal JA, Zoclanclounon YA, Brunner RM, Chen Y, Goldammer T. 2024. Haplotype-resolved and near-T2T genome assembly of the African catfish (Clarias gariepinus). Scientific Data 11(1): 1095.
Xue L, Gao Y, Wu M, Tian T, Fan H, Huang Y, Huang Z, Li D, Xu L. 2021. Telomere-to-telomere assembly of a fish Y chromosome reveals the origin of a young sex chromosome pair. Genome biology 22(1): 203.

## Cookie Preference Center

We use cookies which are necessary to make our site work. We may also use additional cookies to analyze, improve and personalize our content and your digital experience. For more information, see our Cookie Policy.

You may choose not to allow some types of cookies. However, blocking some types may impact your experience of our site and the services we are able to offer. See the different category headings below to find out more or change your settings.

Allow all

## Manage Consent Preferences

### Strictly Necessary Cookies

**Always active**

These cookies are necessary for the website to function and cannot be switched off in our systems. They are usually only set in response to actions made by you which amount to a request for services, such as setting your privacy preferences, logging in or filling in forms. You can set your browser to block or alert you about these cookies, but some parts of the site will not then work. These cookies do not store any personally identifiable information.

### Functional Cookies

These cookies enable the website to provide enhanced functionality and personalisation. They may be set by us or by third party providers whose services we have added to our pages. If you do not allow these cookies then some or all of these services may not function properly.

### Performance Cookies

These cookies allow us to count visits and traffic sources so we can measure and improve the performance of our site. They help us to know which pages are the most and least popular and see how visitors move around the site.

**Targeting Cookies**

These cookies may be set through our site by our advertising partners. They may be used by those companies to build a profile of your interests and show you relevant adverts on other sites. If you do not allow these cookies, you will experience less targeted advertising.

### Cookie List

Clear

Apply Cancel
Consent Leg.Interest

Confirm my choices

Powered by onetrust
