# Supplementary material for: The telomere-to-telomere gapless genome of grass carp provides insights for genetic improvement
Source: Gigascience. 2025 Jun 18;14:giaf059. doi: 10.1093/gigascience/giaf059 (PMC12204074; doi:10.1093/gigascience/giaf059)
Supplement: giaf059_Reviewer_1_Report_original_submission — Kun Wang -- 4/3/2025 [file giaf059_reviewer_1_report_original_submission.pdf]

Reviewer Report

Title: The telomere-to-telomere gapless genome of grass carp provide insights for genetic improvement

Version: Original Submission **Date:** 4/3/2025

Reviewer name: Kun Wang

Reviewer Comments to Author:

This manuscript presents the generation and initial characterization of a telomere-to-telomere (T2T), gapless genome assembly for the economically important grass carp, Ctenopharyngodon idella. The authors utilized a state-of-the-art combination of PacBio HiFi, Oxford Nanopore ultra-long reads, Hi-C, and Illumina sequencing, achieving impressive assembly quality metrics (T2T chromosomes, high BUSCO, QV, and GCI scores). The methods are detailed, validation is thorough, and the data availability statement is clear. While the manuscript is generally strong, a few minor points could further improve its clarity and impact.1. The Introduction states that Mastacembelus armatus is the only fish (at the time of writing) with a chromosome-level T2T assembly. While likely true when drafted, the T2T field moves rapidly. Please double-check if any other fish T2T assemblies (chromosome-level) have been published very recently and update the statement if necessary for accuracy at the time of publication.2. The authors state they used different assembly strategies/tools (Hifiasm, verkko, NextDenovo) and selected the "best assembly" (Hifiasm with HiFi+ONT+Hi-C) as the backbone. The criteria for "best" are not explicitly stated or quantified comparatively.3. Title, 'genome privide insights' should be 'genome provides insights'4. Page 4: The latin name should be italized. This should be checked across the whole manuscript.5. Page 7: The line spacing is not unified.

Level of Interest

Please indicate how interesting you found the manuscript: Choose an item.

Quality of Written English

Please indicate the quality of language in the manuscript: Choose an item.

Declaration of Competing Interests

Please complete a declaration of competing interests, considering the following questions:

- Have you in the past five years received reimbursements, fees, funding, or salary from an organisation that may in any way gain or lose financially from the publication of this manuscript, either now or in the future?
- Do you hold any stocks or shares in an organisation that may in any way gain or lose financially from the publication of this manuscript, either now or in the future?

- Do you hold or are you currently applying for any patents relating to the content of the manuscript?
- Have you received reimbursements, fees, funding, or salary from an organization that holds or has applied for patents relating to the content of the manuscript?
- Do you have any other financial competing interests?
- Do you have any non-financial competing interests in relation to this paper?

If you can answer no to all of the above, write 'I declare that I have no competing interests' below. If your reply is yes to any, please give details below.

'I declare that I have no competing interests

I agree to the open peer review policy of the journal. I understand that my name will be included on my report to the authors and, if the manuscript is accepted for publication, my named report including any attachments I upload will be posted on the website along with the authors' responses. I agree for my report to be made available under an Open Access Creative Commons CC-BY license (http://creativecommons.org/licenses/by/4.0/). I understand that any comments which I do not wish to be included in my named report can be included as confidential comments to the editors, which will not be published.

Choose an item.

To further support our reviewers, we have joined with Publons, where you can gain additional credit to further highlight your hard work (see: https://publons.com/journal/530/gigascience). On publication of this paper, your review will be automatically added to Publons, you can then choose whether or not to claim your Publons credit. I understand this statement.

Yes Choose an item.
