# Supplementary material for: The telomere-to-telomere gapless genome of grass carp provides insights for genetic improvement
Source: Gigascience. 2025 Jun 18;14:giaf059. doi: 10.1093/gigascience/giaf059 (PMC12204074; doi:10.1093/gigascience/giaf059)
Supplement: giaf059_Reviewer_2_Report_original_submission — Li Ren -- 4/6/2025 [file giaf059_reviewer_2_report_original_submission.pdf]

Reviewer Report

Title: The telomere-to-telomere gapless genome of grass carp provide insights for genetic improvement

Version: Original Submission **Date:** 4/6/2025

Reviewer name: Li Ren

Reviewer Comments to Author:

Grass carp (Ctenopharyngodon idella) is the most productive freshwater aquaculture species in China. Each year, a large number of studies are published on its genetic improvement, immunity, and nutrition. The newly reported T2T genome in this study provides a valuable reference for researchers working in these areas, both domestically and internationally. As a data note, this manuscript presents a relatively comprehensive analysis of the newly assembled genome. Compared to previously published grass carp genomes, this version shows notable improvements in quality, particularly in telomeric, centromeric, and other repetitive regions. I recommend acceptance after minor revision.Major Points:1. Sample Information: Clarify the sex (male or female) of the grass carp individual used for genome sequencing.2. Genome Size Discrepancy: The manuscript reports an estimated genome size of 808 Mb but a final T2T assembly size of ~891 Mb. Explain the difference (e.g., k-mer estimation vs. assembly output).3. T2T Chromosome Definition: The statement "44 telomeres... 18 of which were T2T" is unclear, given grass carp has 24 chromosomes. Elaborate on the criteria for designating chromosomes as T2T, as only 18 are labeled as such despite the T2T (gap-free) implication.4. Terminology Consistency: In the Results section, "CT2T" appearsâ€"should this be "CyT2T" as used earlier? Ensure consistent terminology throughout.Minor Points:Formatting:Italicize the Latin name Ctenopharyngodon idella in the Methods section under "Sample collection and sequencing" (e.g., "Short-read libraries of C. idella...").Standardize sequencing depth notation in the Results section (e.g., use "~73Ã—," "~91Ã—," "~185Ã—" consistently instead of mixing "73Ã—" and "91Ã—").Fix general formatting errors: remove double spaces, standardize "HiFi" (not "HIFI"), and adjust the section title "Positive Selection Analysis" to "Positive selection analysis" (capitalize only the first word).Add line numbers to the manuscript for easier review.Grammatical and Clarity Improvements:Revise "Different and high-coverage sequencing reads were used" to "Various high-coverage sequencing reads were used" for smoother phrasing.Correct "The rest genome assembles were used" to "The remaining genome assemblies were used."Add a subject to "After addition of the rDNA arrays and telomere patching..." (e.g., "After we added the rDNA arrays and performed telomere patching...").Simplify "Hi-C chromatin interaction maps exhibited consistent consistency" (redundant) to "Hi-C chromatin interaction maps showed strong consistency" or similar.Change "three genes are associated" to "were associated" for consistent past tense.Precision and Clarity:The "GCI score of 99.9999%" seems overly precise. Round to 99.99% unless the method justifies this level of detail.

Level of Interest

Please indicate how interesting you found the manuscript: Choose an item.

Quality of Written English

Please indicate the quality of language in the manuscript: Choose an item.

Declaration of Competing Interests

Please complete a declaration of competing interests, considering the following questions:

- Have you in the past five years received reimbursements, fees, funding, or salary from an organisation that may in any way gain or lose financially from the publication of this manuscript, either now or in the future?
- Do you hold any stocks or shares in an organisation that may in any way gain or lose financially from the publication of this manuscript, either now or in the future?
- Do you hold or are you currently applying for any patents relating to the content of the manuscript?
- Have you received reimbursements, fees, funding, or salary from an organization that holds or has applied for patents relating to the content of the manuscript?
- Do you have any other financial competing interests?
- Do you have any non-financial competing interests in relation to this paper?

If you can answer no to all of the above, write 'I declare that I have no competing interests' below. If your reply is yes to any, please give details below.

I declare that I have no competing interests

I agree to the open peer review policy of the journal. I understand that my name will be included on my report to the authors and, if the manuscript is accepted for publication, my named report including any attachments I upload will be posted on the website along with the authors' responses. I agree for my report to be made available under an Open Access Creative Commons CC-BY license (http://creativecommons.org/licenses/by/4.0/). I understand that any comments which I do not wish to be included in my named report can be included as confidential comments to the editors, which will not be published.

Choose an item.

To further support our reviewers, we have joined with Publons, where you can gain additional credit to further highlight your hard work (see: https://publons.com/journal/530/gigascience). On publication of this paper, your review will be automatically added to Publons, you can then choose whether or not to claim your Publons credit. I understand this statement.

Yes Choose an item.
